# Multi-omics insights into the role of mitophagy receptor-related genes in glioma prognosis and immune microenvironment remodeling

**DOI:** 10.3389/fimmu.2025.1672678

**Published:** 2025-10-15

**Authors:** Yuheng Xu, Wanqi Weng, Yuanyi Xiong, Jintao Hu, Chunmei Chen, Zhiqiang Peng, Zhaotao Wang

**Affiliations:** ^1^ Department of Neurosurgery, The Second People’s Hospital of Fujian Province (The Second Affiliated Hospital of Fujian University of Traditional Chinese Medicine), Fuzhou, China; ^2^ Department of Anesthesiology, The Second Clinical College of Guangzhou Medical University, Guangzhou, China; ^3^ Institute of Neuroscience, Department of Neurosurgery, The Second Affiliated Hospital of Guangzhou Medical University, Guangzhou, China; ^4^ Department of Stroke Center, The Affiliated Panyu Central Hospital of Guangzhou Medical University, Guangzhou, China

**Keywords:** mitophagy receptors, glioma, immune infiltration, prognosis, IFNAR2

## Abstract

**Background:**

Mitophagy receptor-related genes (MRRGs) orchestrate mitochondrial quality control and may shape glioma progression and immune tolerance, yet their integrated prognostic and immunobiological significance remains unclear.

**Methods:**

We combined WGCNA, single−cell AUCell scoring, and LASSO/Cox modeling across public glioma cohorts to derive and externally validate a 17−gene MRRG risk signature. Multi−omics comparisons (transcriptome, pathway enrichment, mutation, and GWAS association), immune infiltration, and therapy response prediction were performed. Core driver(s) were interrogated by *in vitro* functional assays and *in vivo* xenograft validation.

**Results:**

The MRRG signature robustly stratified overall survival across independent datasets and remained an independent prognostic factor after multivariable adjustment. High−risk tumors exhibited activation of P53 signaling and MAPK signaling pathway, coupled with immunosuppressive remodeling characterized by increased M2−like macrophage infiltration and T cell dysfunction. Integrative analyses highlighted IFNAR2 as a central node; its silencing impaired glioma cell proliferation, invasion, and metastatic potential, while *in vivo* suppression attenuated tumor growth. The model correlated with differential predicted sensitivity to immunotherapy and targeted agents, suggesting potential for precision stratification.

**Conclusion:**

We present and validate a 17−MRRG prognostic model that links mitophagy receptors to glioma immunosuppression and clinical outcome, and identify IFNAR2 as a functional driver. These findings provide a rationale for incorporating MRRG profiling into prognostic assessment and therapeutic decision−making in glioma.

## Introduction

Gliomas represent the most common primary malignant tumors of the central nervous system, encompassing low-grade gliomas (LGG) and glioblastoma (GBM), with high-grade subtypes characterized by aggressive invasiveness and therapeutic resistance (1). Despite continuous advancements in innovative therapeutic strategies including immunotherapy and molecular-targeted therapy, along with optimized standard treatment protocols (e.g., surgery), patient prognosis remains suboptimal (2–4). This underscores the urgent need for establishing precise prognostic evaluation systems and identifying novel biomarkers to advance personalized treatment.

As a critical form of selective autophagy, mitophagy eliminates damaged mitochondria through ubiquitin-dependent (e.g., PINK1/Parkin pathway) or -independent mechanisms, playing a vital role in mitochondrial homeostasis (5). Emerging evidence revealed its pro-tumorigenic properties: OMA1-mediated mitophagy in GBM suppresses cGAS-STING signaling to facilitate immune evasion (6); hypoxia-induced BNIP3 drives metabolic reprogramming in uveal melanoma progression (7); while TMX2-triggered mitophagy enhances hepatocellular carcinoma cell survival (8). These findings suggest mitophagy may promote tumor progression through multidimensional mechanisms involving metabolic reprogramming and immune regulation. Notably, this process is guided by specific receiver proteins like FUNDC1. However, current research predominantly focuses on individual receptors, leaving the holistic regulation network of mitophagy receptors and their crosstalk with the immune microenvironment in gliomas unexplored.

This research thoroughly evaluated the biological characteristics of mitophagy receptor-related genes (MRRG) in gliomas through multi-omics approaches. We pioneered the construction of an MRRG prognostic risk model, validated through multiple cohorts as an independent prognostic indicator. Pan-cancer analyses confirmed its broad applicability in prognostic prediction. Comprehensive immune abundance and functional pathway algorithms also revealed the complicated interaction of MRRG features with the immunosuppressive microenvironment and pro-oncogenic signaling pathways. GWAS genetic association analysis and experimental validation further elucidated the regulatory role of the key gene IFNAR2 in glioma progression. This investigation not only contributed new insights for prognostic assessment in gliomas but also pointed to a novel biomarker for developing targeted therapeutic strategies.

## Materials and methods

### Data source

Transcriptomic features and corresponding clinical data were enrolled from the Cancer Genome Atlas (TCGA) database (https://portal.gdc.cancer.gov/), the GEO database [Home - GEO - NCBI (nih.gov)], the UCSC XENA database (https://xenabrowser.net/datapages/), and the Chinese Glioma Genome Atlas (CGGA) database (http://www.cgga.org.cn).

### Mitophagy receptor gene collection

Through a systematic review of the relevant literature, 16 mitophagy receptor genes were identified (9–22). The complete gene list and supporting references are provided in **Supplementary Table S1**.

### WGCNA calculation

Weighted gene co-expression network analysis (WGCNA) is an algorithm capable of determining gene sets in connection with the co-expression of specific features. The purpose of this investigation has been to utilize this tool to identify the most associated co-expressed genes for mitophagy receptor enrichment scores.

### Extracting and analyzing single-cell data

We have extracted Sc-RNA data from 16,201 glioma cells from the GSE131928 dataset and utilized the Seurat package for calculation (23). Strict qualitative control was first engaged in discarding low-quality and potentially multiple-captured cells and in eliminating cells with abnormal mitochondrial gene content, resulting in the retention of high-quality cells for subsequent analyses. Data were processed for normalization and standardization. Following that, dimensionality reduction and clustering were accomplished, and clustering was performed via the CellMarkers2 website (http://117.50.127.228/CellMarker/index.html) and classical Markers (23, 24). To further investigate mitophagy receptor activity, we scored each cell using the AUCell algorithm and categorized the cells into High and Low-level groups based on median thresholds, and identified differentially expressed genes between the two groups, which were labeled as MDEGs. Eventually, the CellChat package was used to construct a ligand-receptor communication network between cell subtypes.

### The building and valuation of the MRRG risk model

We proceeded with an intersection analysis of MDEGs and WGCNA co-expression module genes, subsequently performing Cox regression analysis on the intersected genes in order to preliminarily discriminate genes associated with prognosis in gliomas (P < 0.05). Moving on, the least absolute shrinkage and selection operator (Lasso) analysis was utilized based on the Glmnet package using a 10-fold cross-validation method to determine the appropriate lambda value and optimize the prognostic gene set. Ultimately, genes with non-zero risk coefficients were chosen as the building blocks of prognostic models, which was identified as the MRRG risk model. The samples were categorized into High-risk and Low-risk groups by calculating a unique risk score for each sample. In addition, we verified the assessment capability of the risk model by various methodological means.

### Meta-analysis

After digging into relevant databases, several external cohorts were acquired and organized. ORs and 95% CIs were calculated for each cohort and meta-analysis using STATA software.

### Functional pathway analysis

DEGs between two MRRG risk groups of TCGA-GBMLGG have been determined using the limma package (Cut off = |Log (FC)| > 0.5, P.value < 0.05). Utilizing the “GSVA” and “ClusterProfiler” packages, we performed pathway analyses on DEGs.

### Assessment of immune infiltration

Utilizing the “IOBR” package, we computed data from seven immune infiltration algorithms, namely CIBERSORT, MCPcounter, EPIC, ESTIMATE, TIMER, Xcell, and quantiseq to comprehensively investigate the impact of MRRG signature on the remodeling of immune infiltration levels in gliomas (25). Furthermore, the enrichment of various leukocyte subtypes in each sample was quantified using the ssGSEA algorithm.

### Immunotherapy sensitivity analysis

To evaluate the potential responsiveness of glioma samples to immunotherapy, TIDE scores were obtained from the TIDE database (http://tide.dfci.harvard.edu/login/). These scores exhibited a strong positive correlation with immunotherapy resistance. Emerging evidence suggests that epithelial-mesenchymal transition (EMT), a critical mechanism underlying tumor metastasis and invasion, also influences immunotherapy sensitivity (26). To explore this further, we extracted the “HALLMARK_EPITHELIAL_MESENCHYMAL_TRANSITION” gene set from the MsigDB (GSEA | MSigDB (gsea-msigdb.org)) and calculated EMT enrichment scores.

### Tumor mutation burden analysis

TMB is an indicator to assess the extent of heterogeneity of the tumor genome. Research has shown that the degree of TMB in gliomas presents a good correlation with the efficacy of immunotherapy, so the TCGAbiolinks and maftools packages were used to estimate the mutation burden (27).

### Drug sensitivity analysis

The Genomics of Drug Sensitivity in Cancer (GDSC) database (http://www.cancerRxgene.org) serves as a comprehensive repository for profiling tumor-specific drug response characteristics (28). To evaluate therapeutic features in gliomas, we employed the oncoPredict package to calculate the half-maximal inhibitory concentration (IC50) values of clinically related agents. Subsequently, we investigated the association between the MRRG risk scores and drug IC50 values.

### GWAS source

Expression quantitative trait locus (eQTL) data for IFNAR2 (accession: eqtl-a-ENSG00000159110) and genome-wide association study (GWAS) summary statistics for brain tumors (accession: ebi-a-GCST90018800) were retrieved from the IEU Open GWAS Project (https://gwas.mrcieu.ac.uk/).

### Instrumental variable identification

Instrumental variables (IVs) were extracted from eQTL data under stringent criteria (SNP: P < 5×10^-6^), followed by linkage disequilibrium (LD) clumping (r² < 0.001, kb = 10,000 kb, clump = TRUE) to ensure independence of IVs. The quantity of SNPs filtered was four, and the F-value of all SNPs was >= 10, meaning that the strength of the IVs was acceptable.

### Mendelian randomization analysis

The TwoSampleMRpackage in R was used to perform a Mendelian Randomization (MR) analysis between instrumental variables (IVs) and outcome variables. The IVW procedure was applied as the principal analytical framework for causal inference.

### Sensitivity analysis and colocalization analysis

Sensitivity analyses included heterogeneity tests (Cochran’s Q statistic), pleiotropy assessments, and Leave-one-out analysis. Bayesian colocalization analysis with coloc R package within +250/-250 kb flanking regions of the eQTL variant, where SNP.PP.H4 > 0.9 defined significant evidence for shared causal variants.

### Patients and sample

Tumor tissues and matched adjacent specimens were obtained from 24 patients undergoing radical resection at Guangzhou Medical University Affiliated Second Hospital (2017–2022). The protocol adhered to institutional/national ethics standards and the Declaration of Helsinki (1964, amended). Ethical approval was granted by the Hospital Ethics Committee (No. 2020-YJS-KS-01). Informed consent was obtained from all participants; waivers for incapacitated subjects were issued based on the noninterventional nature and public health priorities.

### Cell culture

U87 and U251 were obtained from the Cell Bank, Chinese Academy of Medical Sciences, Beijing, China. The cells were cultured in DMEM (Gibco, USA) supplemented with 10% FBS (Gibco, USA) at 37 °C under 5% CO_2_.

### Transfection

Lentiviral shRNA vectors were transduced per the manufacturer’s protocol. Cells were harvested 48h post-transfection for downstream analyses.

### Western blotting

Cellular proteins were lysed and separated via 10% SDS-PAGE. After PVDF membrane transfer, blocking with 5% BSA/TBST was preceded by overnight incubation with primary antibodies included anti-IFNAR2 (Cell Signaling Technology, Danvers, MA, USA; Cat# 53883), anti-GAPDH (Cell Signaling Technology, USA, Cat#2118) (4 °C). Secondary antibody probing (1h, RT) preceded ECL-based target protein detection.

### Cell viability

Cell viability was quantified via CCK-8 assay. Cells (4,000/well) treated in 96-well plates received 10μl CCK-8 solution (Dojindo, Japan). After 2h incubation (37 °C), absorbance at 450nm was measured-results expressed as survival percentage versus blank controls.

### Clonogenic assay

Cells (500/dish) were seeded in 6cm dishes. Post-treatment, colonies formed over 14 days (medium refreshed triweekly). Methanol: acetic acid (3:1) fixation preceded 0.5% crystal violet staining and colony quantification.

### Transwell assay

Cell migration/invasion was assessed using 8-μm pore Transwell chambers. Upper chambers received 20,000 cells (200μL 1% FBS DMEM); lower chambers contained 20% FBS DMEM as a chemoattractant. Matrigel pre-coating defined invasion assays. After 24h, migrated cells were fixed/stained (0.2% crystal violet). Five random fields (100) were counted per replicate. Experiments were performed in triplicate.

### Xenograft model

Female BALB/c nude mice, aged 6 weeks and weighing 18-20g, were obtained from the Second Affiliated Hospital of Guangzhou Medical University (Guangzhou, China), with all animal care protocols approved by the Institutional Animal Care Committee of the same institution, adhering to the People’s Republic of China National Standard GB/T 35892 for laboratory animal welfare. Mice were maintained under specific pathogen−free conditions at temperature 22 ± 2 °C, relative humidity 50-60%, 12 h light/12 h dark cycle (lights on 07:00-19:00), with autoclaved chow and water ad libitum, corncob bedding, and nesting material enrichment. U87 cells (1×10^6^ in 4μl PBS) were stereotaxically injected into the right striatum of female BALB/c nude mice (6 weeks) at coordinates relative to bregma: +0.5mm A/P, +2.0mm M/L, -2.8mm D/V. Mice exhibiting neurological deficits were euthanized. Briefly, all procedures involving live animals were performed under inhalational isoflurane anesthesia (induction 3-4% and maintenance 1.5-2% in 100% oxygen at 1.0 L/min; animals placed on a heated pad). For any terminal blood or tissue collection, animals were first deeply anesthetized (loss of pedal reflex under isoflurane 4%) and then euthanized by gradual−fill CO_2_ asphyxiation at a displacement (fill) rate of 30–40% of chamber volume per minute, immediately followed by a secondary physical method (cervical dislocation) to ensure death, in accordance with AVMA Guidelines (2020) and institutional IACUC approval. No paralytics were used. Maximum tumor volume or humane endpoints triggered immediate euthanasia under the same protocol. Brains were fixed in 4% PFA, paraffin-sectioned coronally. Maximal tumor area was measured with ImageJ; volume calculated as V (a*b2)/2 (a: major axis, b: minor axis). Survival endpoint criteria: immediate sacrifice when moribund, or at 30 days post-injection. Procedures complied with ARRIVE guidelines and IACUC approval.

### Statistical analysis

Statistical analyses were performed using R (v4.4.2) and SPSS 25 (IBM). Before conducting statistical tests, normality (Shapiro-Wilk test) and variance homogeneity (Levene’ s or Brown-Forsythe test) were evaluated. Two-group comparisons employed T-test when parametric assumptions were met, Welch’s t-test for unequal variances, and the Wilcoxon rank-sum test for non-parametric conditions. Multi-group comparisons utilized one-way ANOVA with Tukey’s *post hoc* test for normally distributed data, and the Kruskal-Wallis test with Dunn’s *post hoc* correction for non-parametric data. The Benjamini-Hochberg (BH) procedure controlled the false discovery rate (FDR). Normally distributed data are expressed as mean ± standard deviation (SD), while non-normal data are reported as median [interquartile range (IQR)]. Effect sizes (e.g., HR, correlation coefficients) and confidence intervals are provided throughout. All expression datasets underwent stringent quality control; only samples with technical errors were excluded. Statistical significance was defined as two-tailed P < 0.05 or FDR-adjusted Q < 0.05, with emphasis on effect size and confidence interval interpretation.

## Result

### Identification of co-expressed genes with mitophagy receptor enrichment scores utilizing WGCNA analysis

**Figure 1:**
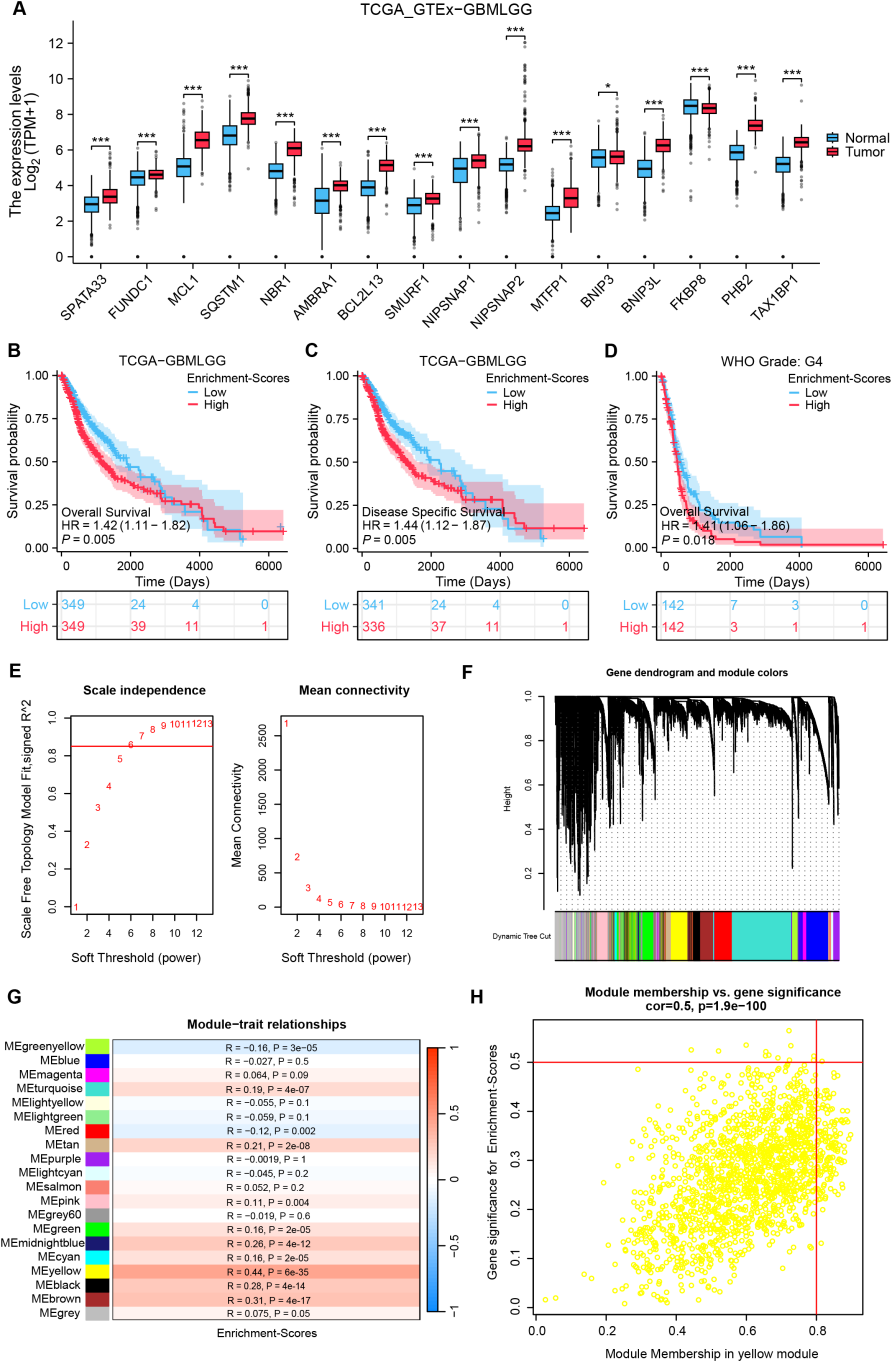
Detection of co-expressed genes with mitophagy receptor enrichment scores utilizing WGCNA analysis. **(A)** Comparison analysis of mitophagy receptor in glioma and normal tissues. (test: [Wilcoxon rank−sum test], n(Tumor)= [1157], n(Normal)= [689]). **(B–D)** KM curves for OS in high vs low mitophagy receptor enrichment score groups across three cohorts. [HR and 95% CI from univariate Cox, Cohorts: **(B)** TCGA (n = 698); **(C)** CGGA (n = 325)]. **(E)** Soft threshold screening for WGCNA network construction: the red line denotes the optimal soft threshold (Soft threshold = 0.85). **(F)** Network diagram of module construction of WGCNA. **(G)** Heatmap depicting correlations between gene modules and enrichment scores. **(H)** Scatterplot of correlation between ME yellow module genes and enrichment scores. *P < 0.05, ***P < 0.001, HR, hazard ratio; CI, confidence interval.

**Figure 1A** revealed significantly altered expression of 16 mitophagy receptor genes in glioma versus normal tissues (P < 0.05). To preliminarily evaluate the role of mitophagy receptors in gliomas, we have calculated TCGA-GBMLGG enrichment scores based on these genes and stratified the samples into High and Low groups using mid-score thresholds. KM analyses displayed a strong association between high enrichment scores and adverse prognosis in glioma patients (all, HR > 1, P < 0.05, **Figures 1B–D**). Next, the clustered abnormal samples were eliminated, and the remaining samples were subjected to WGCNA analysis, in which optimal connectivity of the data and the presentation of a power-law distribution were secured when the soft-threshold parameter was 6 and the R2 > 0.85 (**Figures 1E, F**). For gene module clustering, a deep split of 2 was chosen, and a similarity threshold of 0.2 was applied to ensure module independence, resulting in the identification of 20 gene modules. Finally, as shown in **Figures 1G, H**, the MEyellow module, comprising 1576 genes, exhibited the highest correlation with mitophagy receptor enrichment scores (R = 0.44, P = 6×10^-^³^5^).

### Single-cell fractionation identification and calculation of mitophagy receptors AUCell scores

**Figure 2:**
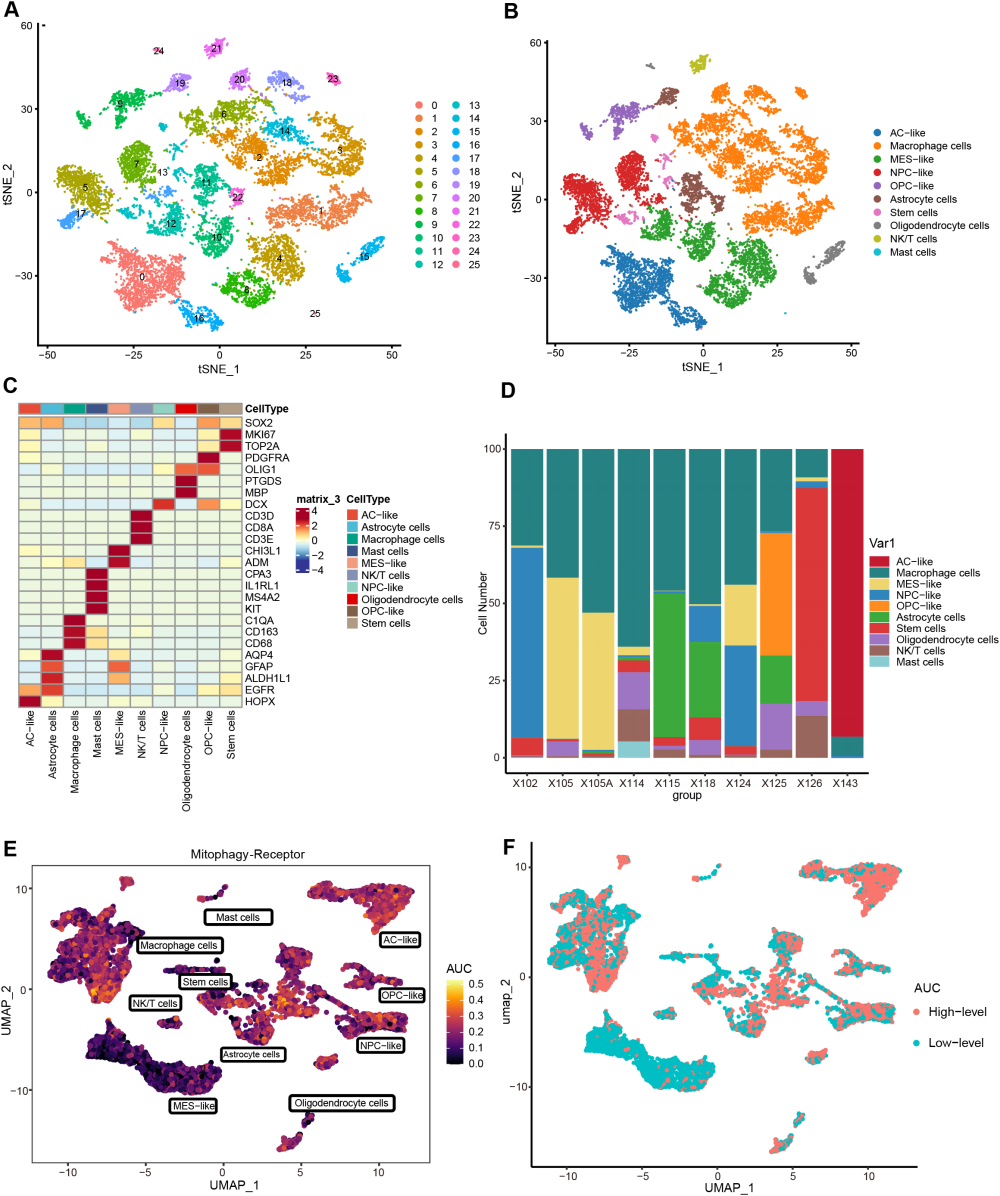
Determining single-cell subpopulations and performing AUCell analysis. **(A)** t-SNE visualization of 26 clusters. **(B)** Cell types are annotated into 10 categories, with specific colors for each cluster. **(C)** Heatmap of signature gene expression across cellular subclusters. **(D)** Proportional distribution of cell types in 10 samples. **(E)** AUCell scores assessing the abundance of mitophagy receptor activity in individual cells. **(F)** Distribution of cells with high and low-level AUCell scores. (Threshold = median of AUCell scores).

To investigate the impact of mitophagy receptors on gliomas at the single-cell level, we have performed comprehensive single-cell analyses. Following stringent quality control, high-quality cells were reserved for downstream analysis. PCA revealed no significant heterogeneity in cell cycle scores across the dataset (**Supplementary Figure S1A**). No batch effect treatment was applied in this investigation because this dataset had been subjected to batch effect reduction prior to extraction. Subsequent dimensionality reduction and clustering partitioned the cells into 26 distinct subpopulations (**Figure 2A**, **Supplementary Figure S1B**). Based on canonical marker genes, these subpopulations were annotated into 10 cell types: AC-like (EGFR, HOPX), Macrophage cells (C1QA, CD163, CD68), MES-like (CHI3L1, ADM), NPC-like (DCX), OPC-like (PDGFRA, OLIG1), Astrocyte cells (AQP4, GFAP, ALDH1L1), Stem cells (SOX2, TOP2A, MKI67), Oligodendrocyte cells (PTGDS, MBP), NK/T cells (CD3D, CD8A, CD3E), and Mast cells (CPA3, IL1RL1, MS4A2, KIT). The annotated clusters were visualized using UMAP and t-SNE (**Figure 2B**, **Supplementary Figure S1C**), while a heatmap highlighted the expression levels of key markers for each cluster (**Figure 2C**). **Figure 2D** illustrated the cellular composition across samples. To assess mitophagy receptor activity, AUCell scores were calculated for each cell. Notably, tumor-associated subpopulations (AC/OPC/NPC-like cells) exhibited higher AUCell scores, suggesting a potential role of mitophagy receptors in tumor heterogeneity (**Figure 2E**). Cells were stratified into High and Low-level groups based on the median AUCell threshold, followed by identifying 1037 MDEGs between the groups (Cut off = |Log2FC| > 0.5, P.val < 0.05) (**Figure 2F**).

### Cell-chat analysis of cell subpopulations

**Figure 3:**
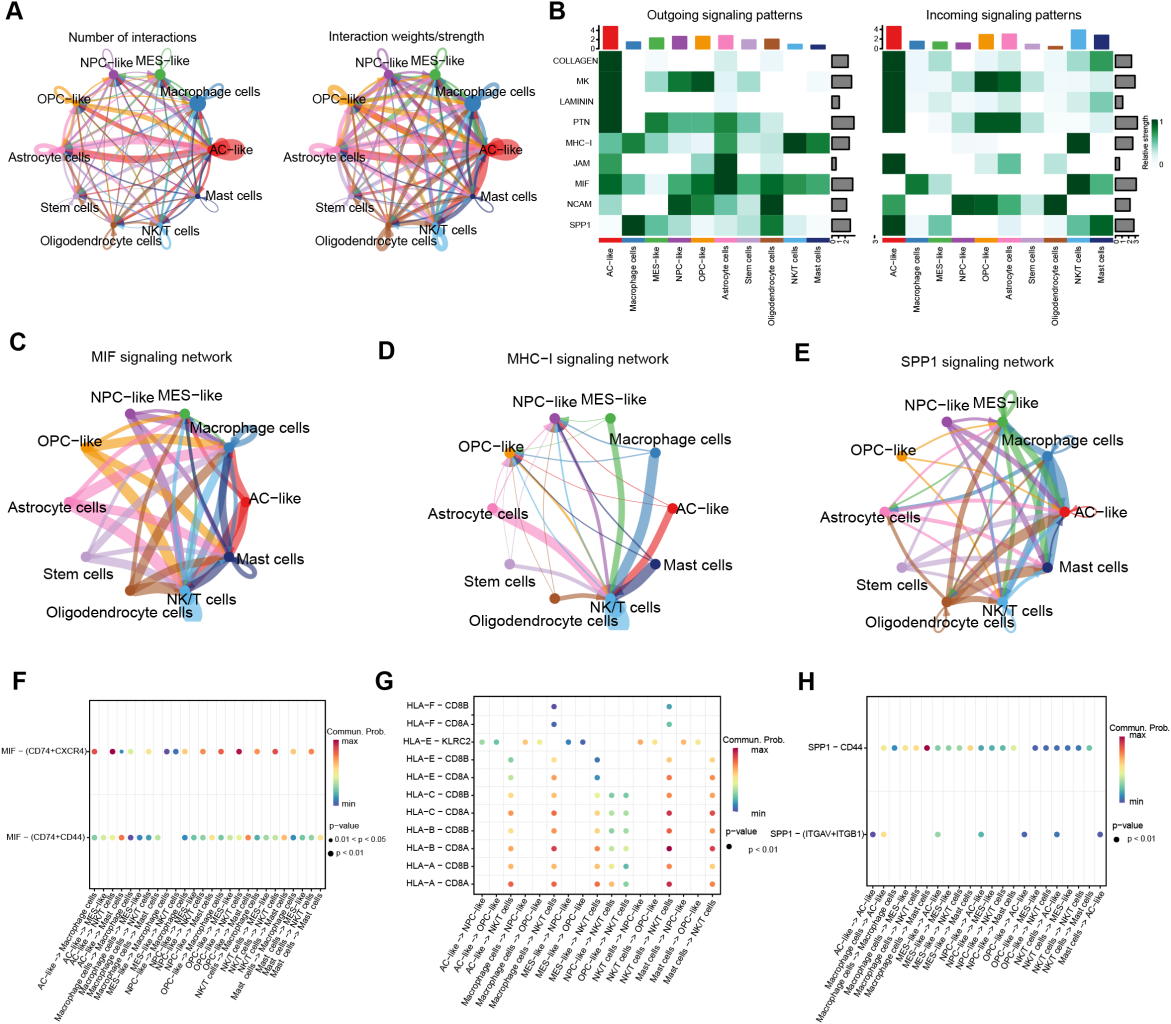
Cell-Chat analyses. **(A)** circle plot depicting the number of communications between cell subclusters and the total communication intensity. **(B)** Heatmap of the top 9 signaling pathway activations. **(C–E)** Circle plots to further visualize the communication between cell subclusters of the MIF, MHC-I, and SPP1 pathways. **(F–H)** Bubble plots of ligand-receptor interactions between immunologic and malignant cell clusters (bubble size: P-value; color intensity: interaction weight).

To decipher the intricate signaling network within the tumor microenvironment, we performed cell-cell communication analysis using the Cell-chat algorithm. As depicted in **Figure 3A**, a complex interaction network was observed among cellular subpopulations, while **Figure 3B** demonstrated the most active signaling pathways. Given the vital role of leukocytes in microenvironment regulation, we focused on MIF, MHC-I, and SPP1 signaling systems. **Figures 3C, F**, and **Supplementary Figure S2A** revealed that MIF signals were predominantly secreted by AC/NPC/OPC-like, transmitting signals through CD74-CXCR4 receptor complexes to NK/T cells, and Macrophage cells, with partial signaling via CD74-CD44 to Mast cells. Prior evidence indicated the MIF-CD74/CXCR4 signaling axis drives brain tumor progression through comprehensive mechanisms: suppressing pro-inflammatory microglial differentiation while facilitating tumor vascular network formation (29, 30). Further analysis indicated that MHC-I-mediated HLA-CD8 interactions constituted an immunoregulatory network among tumor cells, macrophages, and NK/T cells (**Figures 3D, G**, **Supplementary Figure S2B**). Notably, macrophages coordinately regulated NK/T cells, AC-like cells, and MES-like cells through SPP1 secretion and SPP1-CD44 signaling activation (**Figures 3E, H**, **Supplementary Figure S2C**). Emerging evidence suggests that SPP1+ tumor-associated macrophages (TAMs) promote tumor proliferation by inducing T cell exhaustion and epigenetic remodeling (31–34).

### The novel MRRG risk model showed notable prognostic predictive capabilities for gliomas

**Figure 4:**
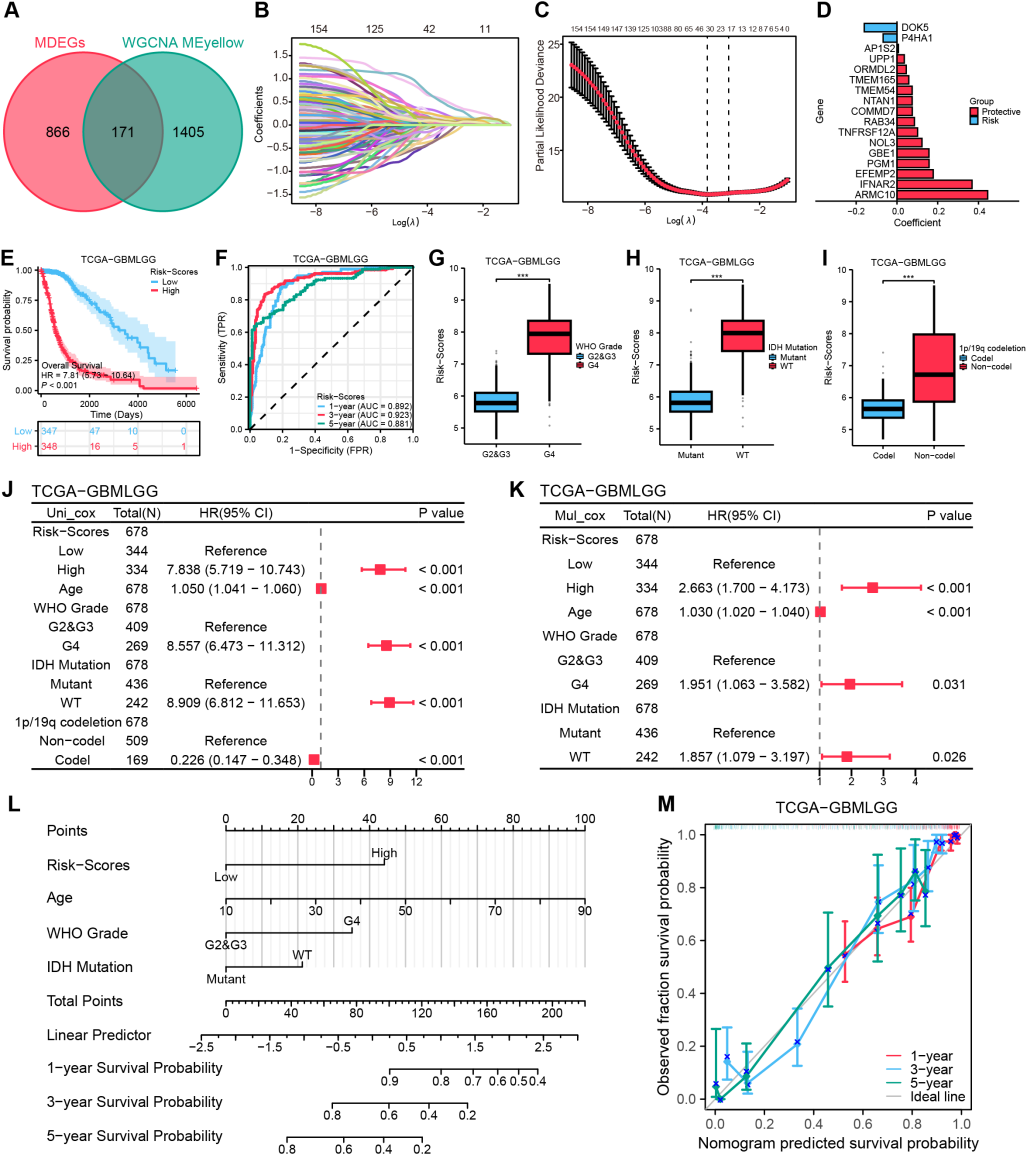
Establishing a risk model based on MRRG traits. **(A)** Venn diagram intersecting MDEGs with ME-yellow module genes. **(B)** Lasso coefficient trajectory plot. **(C)** Lasso coefficient visualization (left dashed line: optimal λ.min; right: λ.1se within 1 standard error). **(D)** Bivariate histogram presenting the key compositions of the MRRG risk model. **(E)** KM curves for OS in the two MRRG risk groups. **(F)** Time-dependent ROC curves of risk scores. **(G–I)** Boxplots showing higher MRRG risk scores in unfavorable phenotypes: **(G)** WHO grade, **(H)** IDH mutation, **(I)** 1p/19q codeletion (Test: Wilcoxon rank sum test). **(J, K)** Forest plots to visualize the outcomes of Cox regressions. **(L)** A nomogram plot integrating clinical parameters. **(M)** 1,3,5-year calibration curves for the nomogram. *** P < 0.001, HR, hazard ratio; CI, confidence interval.

The intersection of WGCNA-MEyellow module genes (n = 1,576) with MDEGs (n = 1,037) yielded 171 hub genes (**Figure 4A**). Subsequently, 163 genes were preliminarily filtered to correlate with the prognosis of gliomas (P < 0.05). Following the systematic consolidation of data on TCGA-GBMLGG, Lasso regression analysis was ultimately implemented, and 17 non-zero coefficient genes were scanned to develop the multigene risk score model, labeled as the MRRG risk model (**Figures 4B–D**). The MRRG signature gene expression of the cell subspecies is presented in **Supplementary Figures S3**, **S4**. The formula for computing the risk score was as follows: Risk-Scores = 0.445 * ARMC10 + 0.369 * IFNAR2 + 0.178 * EFEMP2 + 0.158 * PGM1 + 0.157 * GBE1 + 0.123 * NOL3 + 0.101 * TNFRSF12A + 0.086 * RAB34 + 0.075 * COMMD7 + 0.075 * NTAN1 + 0.075 * TMEM54 + 0.058 * TMEM165 + 0.046 * ORMDL2 + 0.036 * UPP1 + 0.007 * AP1S2 - 0.068 * P4HA1 - 0.160 * DOK5. As demonstrated in **Figure 4E**, the KM survival curve represented a significant difference in overall survival (OS) between the two risk groups (HR = 7.81,95%CI [5.73-10.64], P < 0.001). **Figures 4F–I** highlighted the strong prognostic predictive capability of the MRRG risk scores not only but also the strong positive association with the malignancy of the tumors.

To establish the prognostic significance of the MRRG risk score, multivariate Cox regression has established the MRRG risk score as an independent prognostic component of TCGA-GBMLGG along with other clinical elements (**Figures 4J, K**, **Supplementary Table S2**). Detailed results were Risk-Scores (HR = 2.663, 95%CI [1.700-4.173], P < 0.001), Age (HR = 1.030, 95%CI [1.020-1.040], P < 0.001), WHO Grade (G2&G3 vs. G4, HR = 1.951, 95%CI [1.06.-3.582], P = 0.031), IDH Mutation (HR = 1.857, 95%CI [1.079-3.197], P = 0.026). A nomogram containing the above independent prognostic components was further developed to enhance the overall predictive capacity. The nomogram adopted a cumulative integral pattern to predict the prognosis of gliomas, with higher scores pointing to a poorer prognosis (**Figure 4L**). The nomogram (C-index = 0.839, 95%CI [0.827-0.850]) constructed based on the above factors validated the predictive accuracy by calibration curves (**Figure 4M**).

### Validation of the novel MRRG risk model

**Figure 5:**
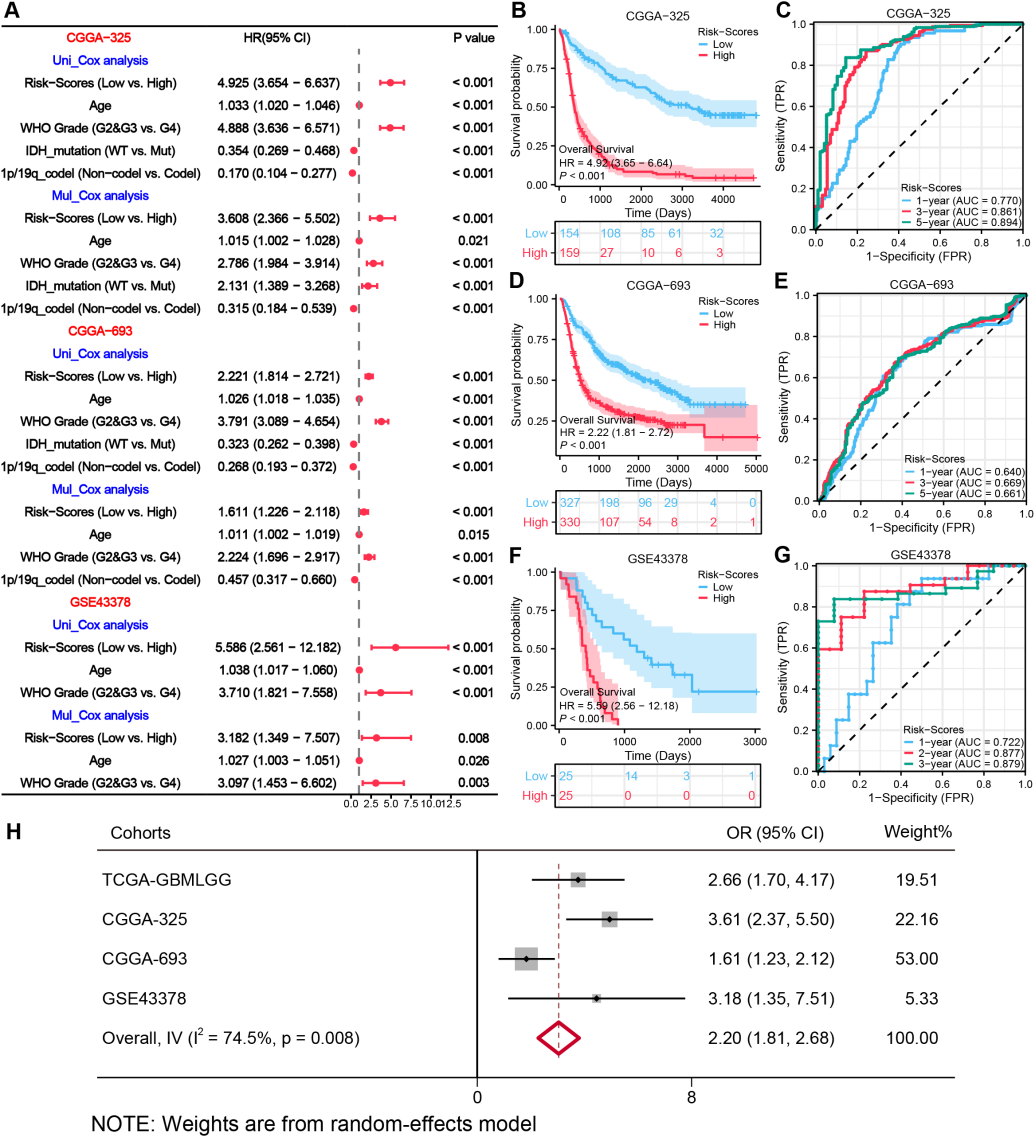
External validation of the MRRG risk model. **(A)** Cox forest plots of external glioma cohorts. **(B, D, F)** KM OS curves (univariate Cox) for risk groups in **(B)** CGGA−325 (n= [325]), **(D)** CGGA−693 (n= [693]), **(F)** GSE43378 (n= [50]) **(C, E, G)** Time-dependent ROC plots of three external cohorts, **(C)** CGGA-325, **(E)** CGGA-693, **(G)** GSE43378. **(H)** Meta-forest plots spanning four cohorts.

To validate the generalizability of the model, we have utilized three external datasets, for validation. Survival analyses revealed significantly lower OS (all, HR > 1, P < 0.001) in the High-risk group across all sets of data (**Figures 5B, D, F**). The 1-, 3-, and 5-year AUC ratings of the risk scores in CGGA-325 and CGGA-693 datasets were CGGA-325 (0.770, 0.861, 0.894) and CGGA-693 (0.640, 0.669, 0.661). The 1-, 2-, and 3-year AUC ratings of the risk scores in GSE43378 were (0.722, 0.877, 0.879) (**Figures 5C, E, G**). The further Cox regression of risk scores across the three datasets consistently demonstrated that Risk-scores was an independent prognostic element (**Figure 5A**). The meta-analysis integrating four cohorts further confirmed the stability of the MRRG model, with a pooled OR of 2.20 (95% CI 1.81-2.68; **Figure 5H**).

### Functional pathway analysis

**Figure 6:**
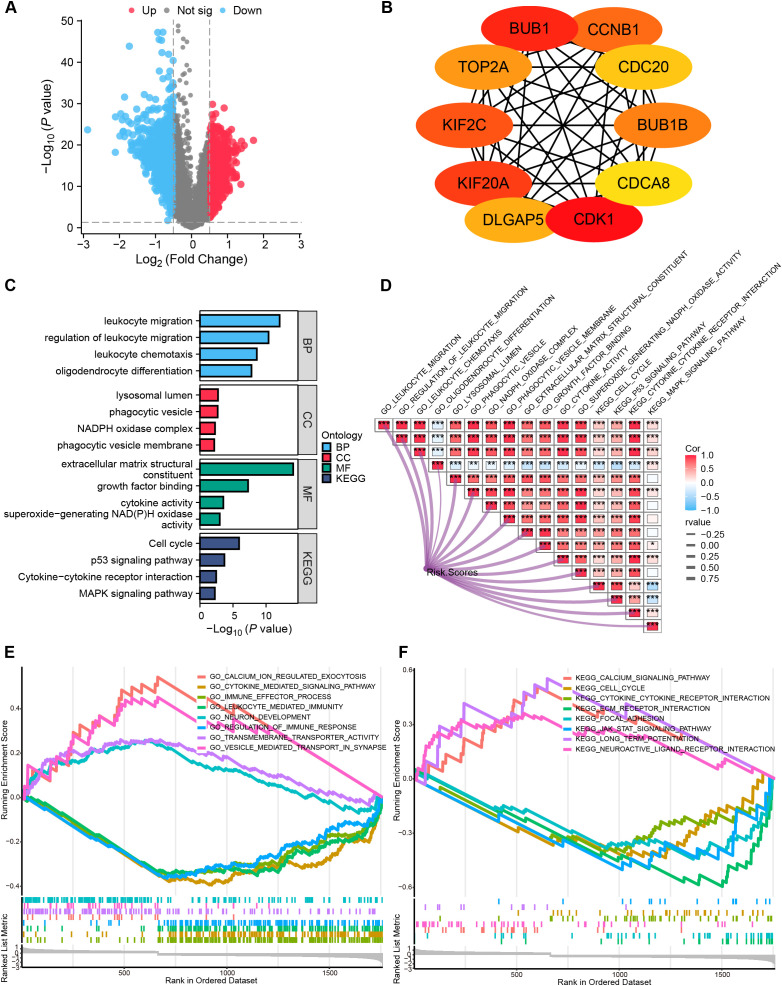
Functional pathway analyses. **(A)** Volcano plot depicting the allocation of DEGs, with red dots depicting notably up-regulated DEGs and blue dots representing notably down-regulated DEGs. **(B)** Top 10 hub genes of the DEGs-based PPI network. **(C)** Histograms highlighting the GO and KEGG pathways enriched by DEGs. **(D)** Correlation network heatmap demonstrating the correlation of risk scores with the enriched pathways (red: positive correlation; blue: negative correlation). **(E, F)** GSEA enrichment analysis outcome. *P < 0.05, ***P < 0.001.

Analysis of the TCGA-GBMLGG cohort identified 1,787 DEGs (|log2FC| > 0.5, P.val < 0.05, **Figure 6A**). Hub genes were characterized via PPI analysis utilizing the Maximal Clique Centrality (MCC) algorithm in Cytoscape (**Figure 6B**). Functional analyses revealed: Biological processes (BP): immune-related pathways, including leukocyte migration, regulation of leukocyte migration, leukocyte chemotaxis, and oligodendrocyte differentiation. Cellular components (CC): lysosomal lumen (implicated in mitophagy), phagocytic vesicle, NADPH oxidase complex, and phagocytic vesicle membrane. Molecular functions (MF): extracellular matrix structural constituent, growth factor binding, cytokine activity, and superoxide-generating NAD (P) H oxidase activity. KEGG pathways: Cell cycle, p53 signaling, cytokine-cytokine receptor interactions, and MAPK signaling pathway (**Figure 6C**). The MRRG risk scores exhibited remarkable positive correlations with these pathways (all, P < 0.001; **Figure 6D**).

GSEA indicated suppression of immune activation pathways in High-risk gliomas (P.adj < 0.05, FDR < 0.25), including JAK-STAT signaling pathway, immune effector process, regulation of immune response, and leukocyte-mediated immunity (**Figures 6E, F**, **Supplementary Table S3**), suggesting an immunosuppressive microenvironment.

### Reshaping of the immunological microenvironment by MRRG signature

**Figure 7:**
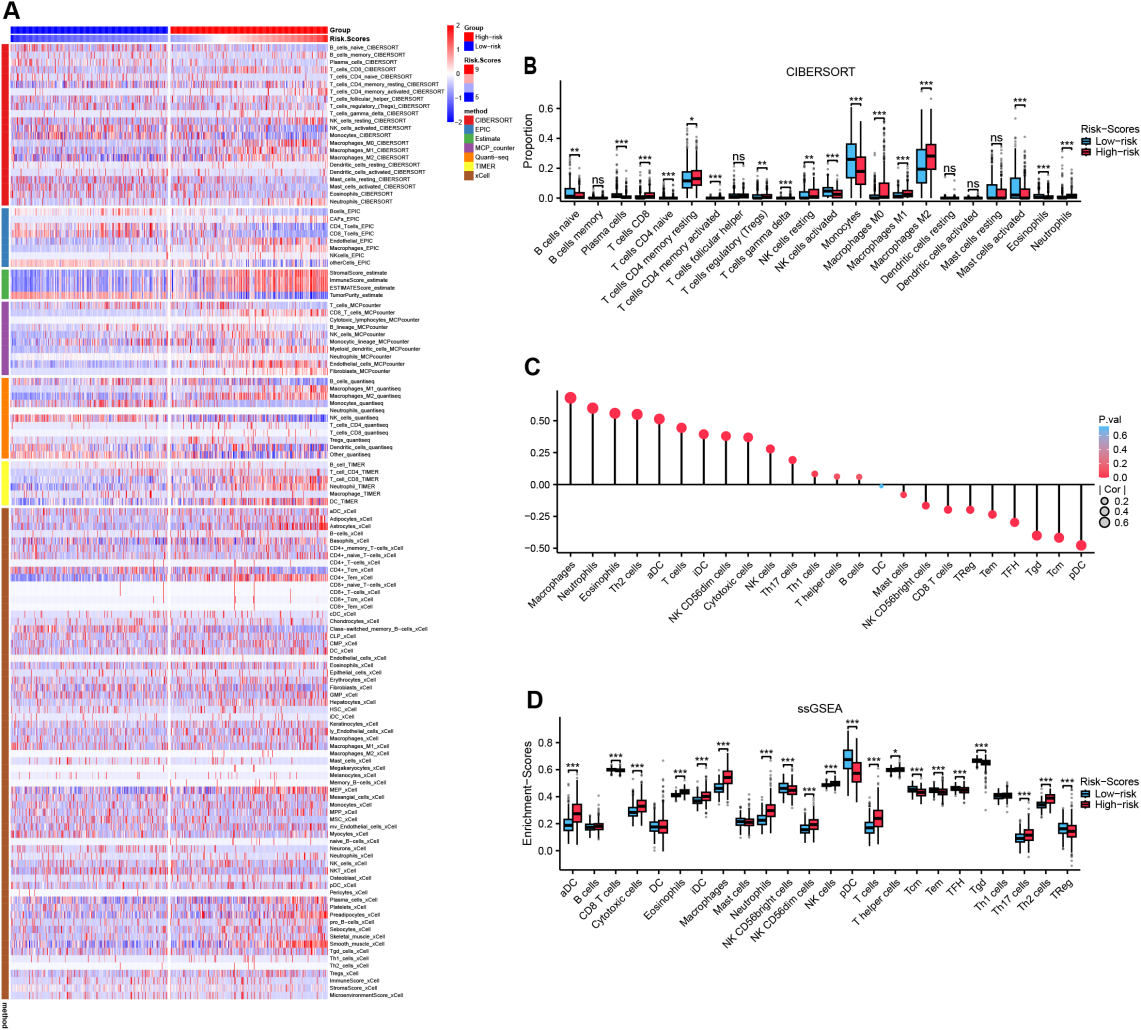
Reshaping of the immunological microenvironment by MRRG signatures. **(A)** Multiple immunization algorithms emphasizing heterogeneity of immune infiltrates across two MRRG risk groups. **(B)** Subgroup comparison plot of leukocyte subtype fractions in two MRRG risk groups (Test: Wilcoxon rank sum test). **(C)** Bubble plot of correlation between risk scores and leukocyte subtype enrichment abundance. **(D)** Subgroup comparison plot of leukocyte subtype enrichment in two MRRG risk groups (Test: Wilcoxon rank sum test). *P < 0.05, **P < 0.01, ***P < 0.001, ns not significant.

A comprehensive evaluation of immune microenvironment signatures using seven distinct algorithms consistently revealed significantly elevated tumor-associated macrophage (TAM) infiltration and stromal scores, coupled with reduced tumor purity in the High-risk group (**Figure 7A**). Quantitative analysis of leukocyte subpopulations via Cibersort demonstrated increased proportions of M0/M1/M2 macrophage subtypes (with M2 anti-inflammatory subtype predominating) alongside increased resting CD4+ T cells, resting NK cells, both of which are immunosuppressive cell subpopulations in the High-risk group (all, P<0.05, **Figure 7B**, **Supplementary Figure S5**). ssGSEA analysis indicated a strong positive correlation between MRRG risk scores and macrophage infiltration (R = 0.680, P < 0.001), whereas a significant negative correlation was observed with plasmacytoid dendritic cell (pDC), a leukocyte subset critical for maintaining immunocompetence (R = -0.477, P < 0.001, **Figure 7C**). Comparative analysis substantiated markedly elevated macrophage enrichment levels and diminished immune active cells activity like pDC, Treg, Tcm, Tem, TFH, Tgd, CD + 8T, and NK CD56 bright in the High-risk group (all, P<0.001; **Figure 7D**). These observations imply that MRRG signature orchestrates an immunosuppressive microenvironment by suppressing leukocyte activation and modulating the differentiation of pro-tumorigenic TAM subsets, ultimately promoting immune evasion and therapeutic resistance in High-risk gliomas.

### Crosstalk between MRRG signature and glioma immunotherapy

**Figure 8:**
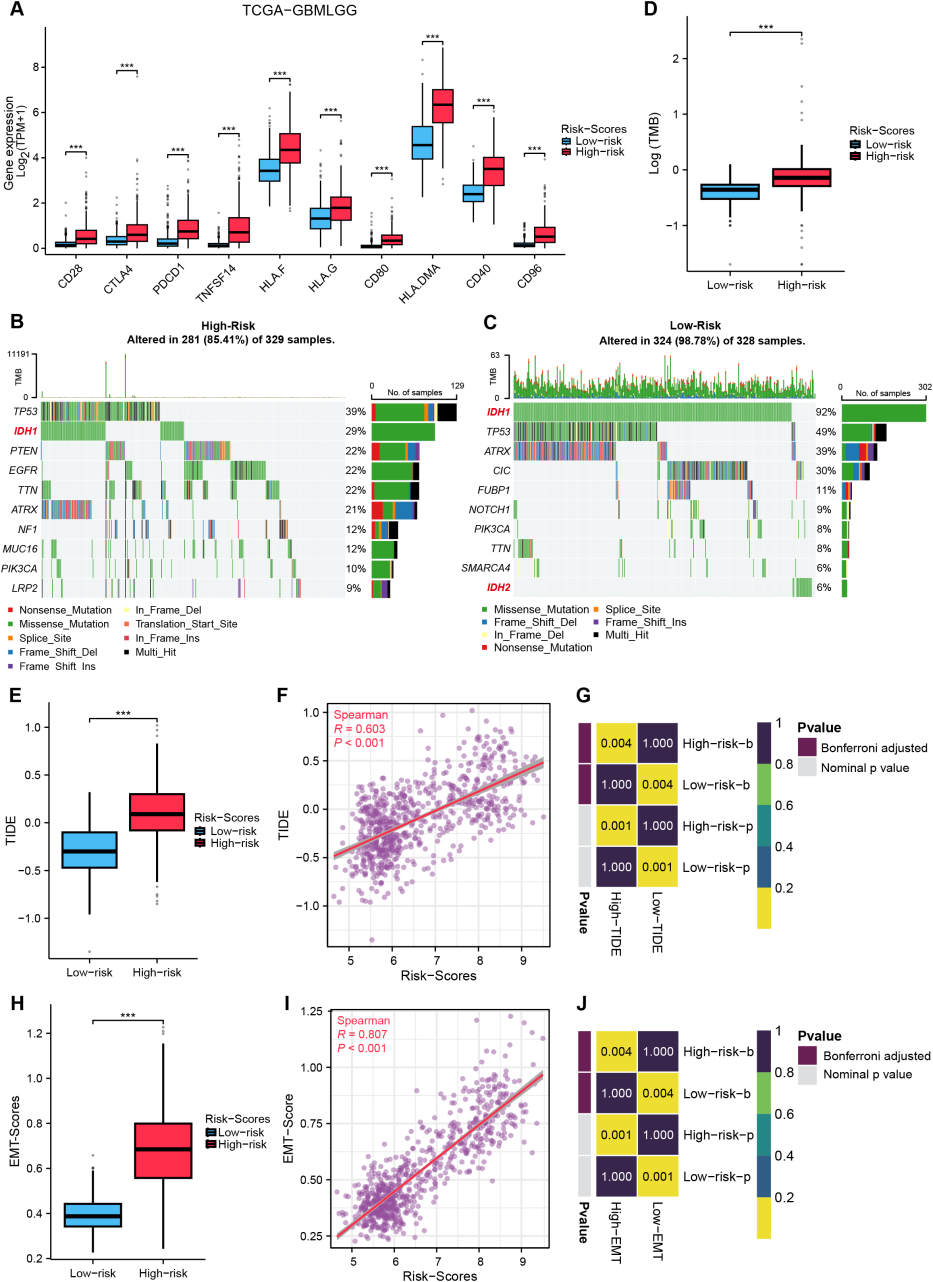
Crosstalk between MRRG signature and gliomas immunotherapy. **(A)** ICP gene expression in two MRRG risk groups (Test: Wilcoxon rank sum test). **(B, C)** Waterfall plots representing TMB in two MRRG risk groups (B-Test: Wilcoxon rank sum test). **(D)** Comparison plot of TMB across different subgroups. **(E–G)** TIDE showed a significant positive correlation with MRRG risk scores (E-Test: T test). **(H–J)** EMT scores strongly correlated with MRRG risk scores (H-Test: Welch t’ test). ***P < 0.001.

Comparative analysis of ICP expression profiles between risk subgroups revealed significant upregulation of 10 critical checkpoints in the High-risk group (**Figure 8A**). The MRRG risk scores demonstrated strong positive correlations with these checkpoint expressions (**Supplementary Table S4**), suggesting enhanced immune tolerance and immunosuppressive microenvironment in high-MRRG-score patients. TMB was significantly elevated in the High-risk group (**Figure 8D**). Notably, IDH1/2-mutant gliomas (favorable prognostic subtype) were significantly enriched in the Low-risk group (**Figures 8B, C**). These outcomes reinforced that TMB is a negative predictor of glioma immunotherapy response. Evaluations using TIDE and EMT scoring systems revealed significantly higher TIDE (**Figure 8E**) and EMT scores (**Figure 8H**) in the High-risk group, both showing strong correlations with MRRG risk scores (**Figures 8F, G, I, J**). These findings indicated that High-risk tumors exhibited enhanced immune evasion capacity and immunotherapy insensitivity.

### Drug sensitivity analysis

**Figure 9:**
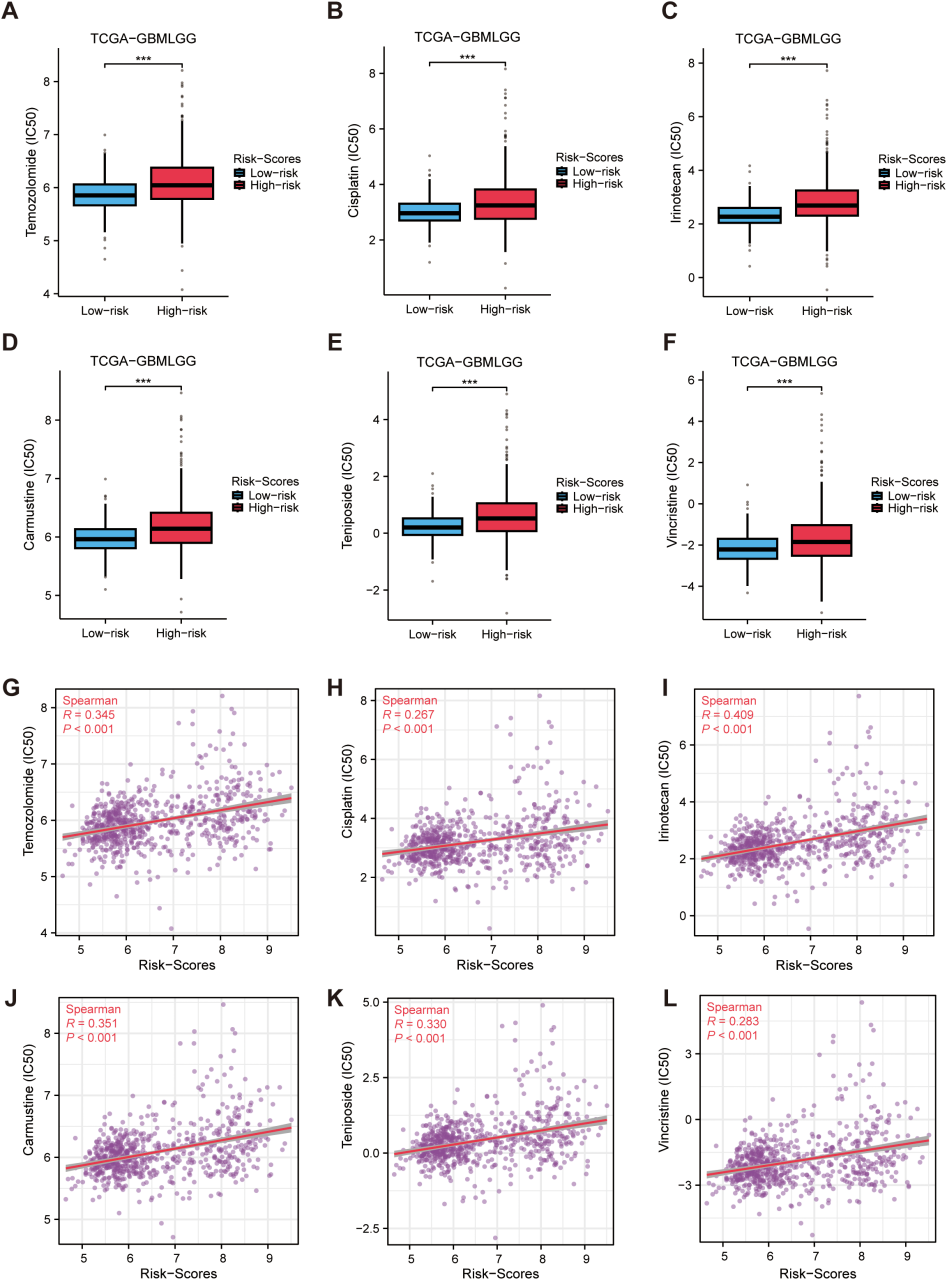
Drug sensitivity analysis. **(A–F)** Differences in predicted drug susceptibility (IC50) to selected agents between high and low MRRG risk groups. **(G–L)** Spearman correlations between MRRG risk scores and predicted drug response metrics for the same agents (asterisks for BH FDR<0.05). ***P < 0.001.

By evaluating immunotherapy sensitivity in patients of various levels of MRRG signature, this study further assessed chemotherapeutic responses in glioma patients. High-risk gliomas exhibited elevated IC50 values for six chemotherapeutics (P < 0.05, **Figure 9**), indicating reduced therapeutic efficacy and enhanced drug resistance potential in the High-risk cohort.

### Mendelian randomization analysis

**Figure 10:**
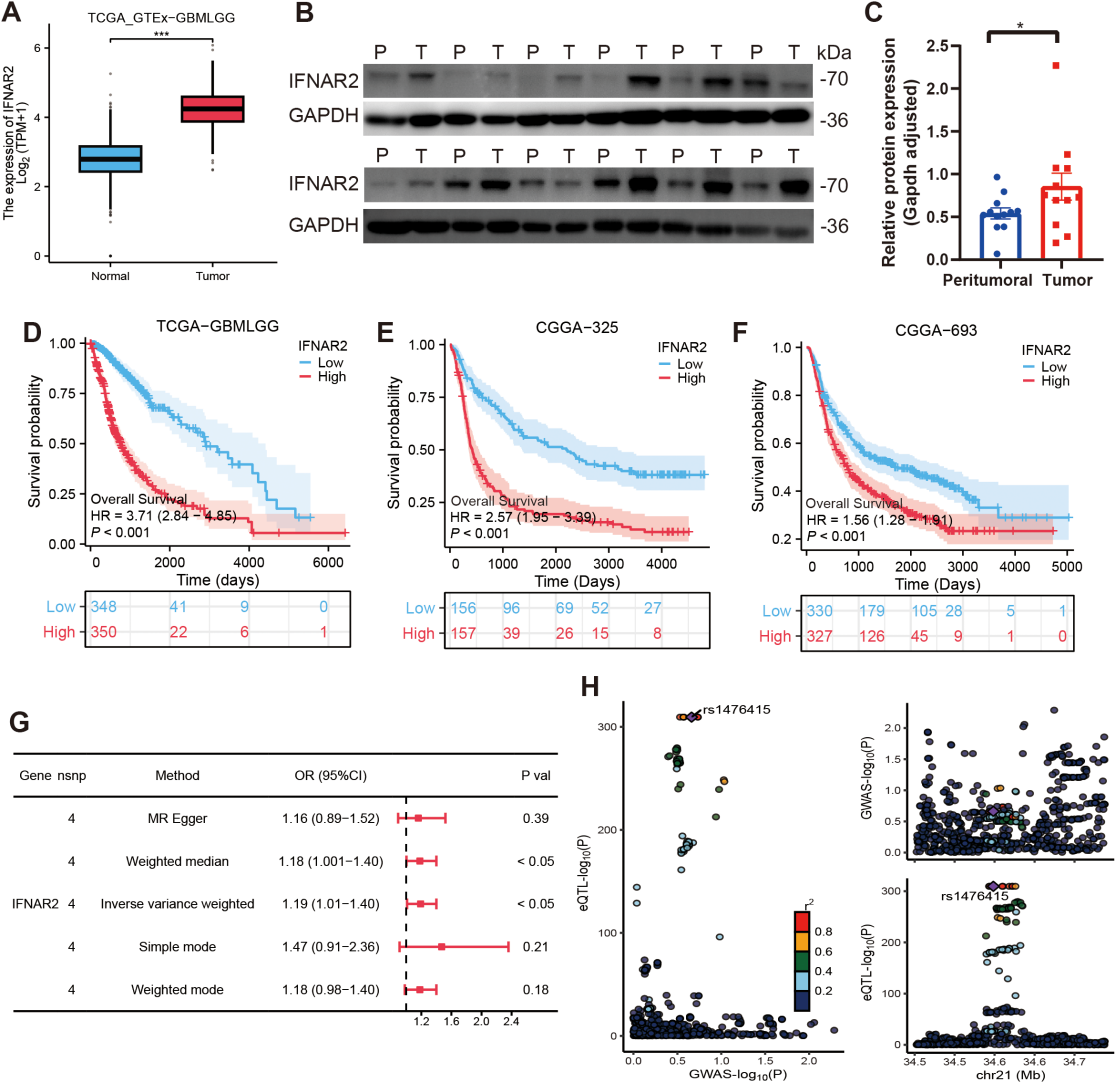
Comprehensive analyses of IFNAR2. **(A–C)** IFNAR2 expression is remarkably elevated in tumor tissues (Test: Wilcoxon rank sum test). A: n (normal)= 1157, n (tumor) = 689; **(B, C)** n (peritumoral) = 12, n (tumor) = 12. **(D–F)** IFNAR2 is linked to an unfavorable prognosis in gliomas. **(G)** Forest plot of MR analysis findings. **(H)** Colocalization analysis results of IFNAR2 and brain tumors. ***P < 0.001.

Given the unique biological role of IFNAR2, a critical component of the MRRG risk model in mitophagy and glioma progression, we have conducted comprehensive multi-omics investigations. Transcriptomic analysis revealed significant upregulation of IFNAR2 mRNA in glioma tissues (P < 0.001), and the corresponding results were also reflected in 12 pairs of tumor tissues and peritumoral tissues from glioma patients (**Figures 10A–C**). KM analyses across glioma cohorts demonstrated markedly reduced OS in IFNAR2-high patients (all, HR > 1, P < 0.001, **Figures 10D–F**). Cox regression analysis confirmed IFNAR2 as an independent prognostic risk element (HR = 1.819, 95%CI [1.119-2.957], P = 0.016; **Supplementary Table S5**). MR analysis (exposure: IFNAR2; outcome: brain tumors) revealed a causal association via the IVW method (OR = 1.19, 95%CI [1.01-1.40], P < 0.05, **Figure 10G**). Sensitivity analyses substantiated robustness: no significant heterogeneity (Cochran’s Q P = 0.60), horizontal pleiotropy (MR-Egger intercept P = 0.86, MR-PRESSO P = 0.608), or influential outliers (leave-one-out analysis; **Supplementary Figure S6**) were detected. Colocalization analysis identified rs1476415 (within -/+ 500kb of IFNAR2) meeting stringent colocalization criteria (SNP.PP.H4 > 0.9), suggesting this locus may regulate IFNAR2 expression to mediate brain tumor pathogenesis (**Figure 10H**).

### The influence of low IFNAR2 expression on the pathobiology of glioma

**Figure 11:**
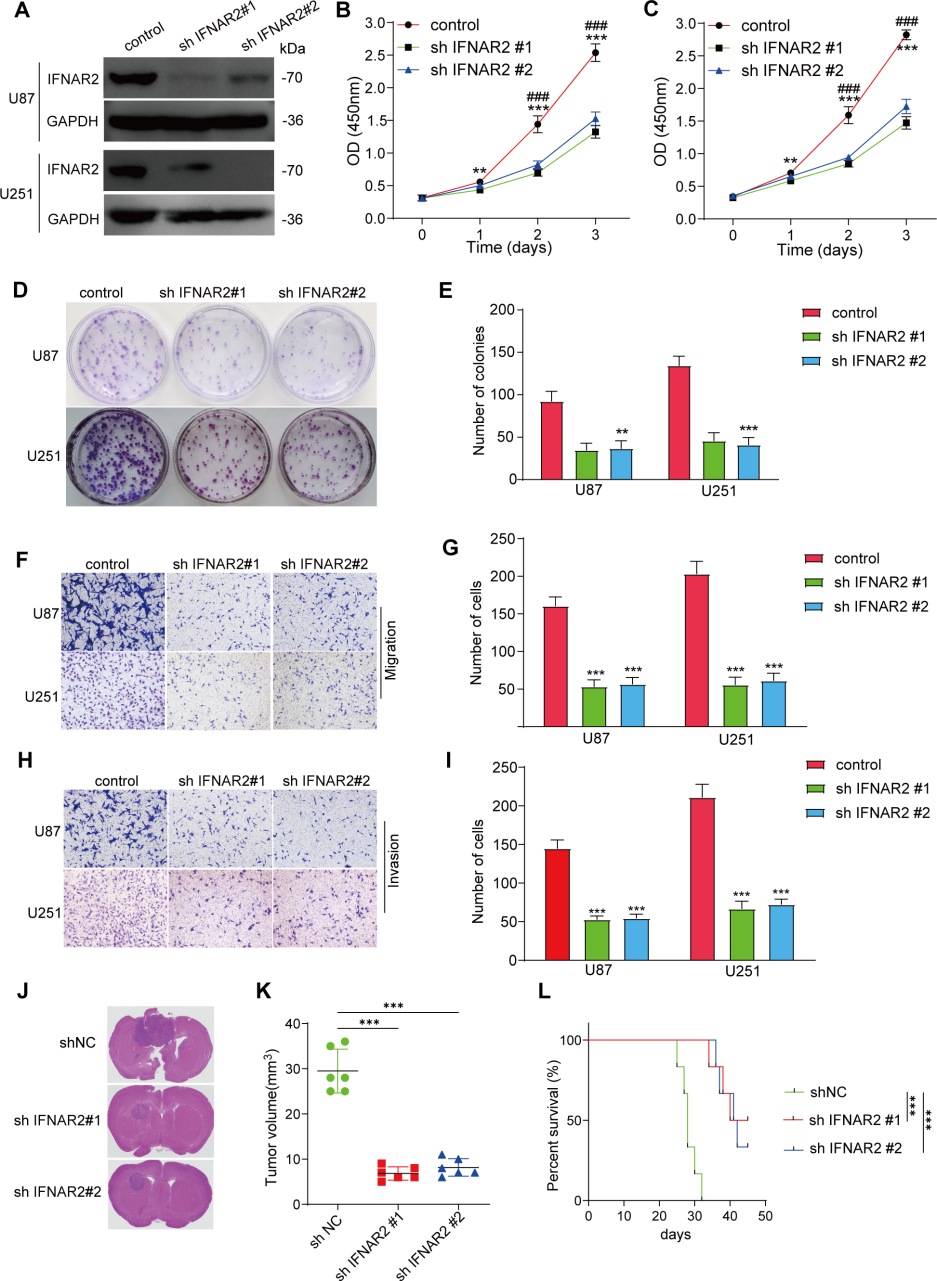
IFNAR2 knockdown inhibits glioma pathobiology. **(A)** Western blot of IFNAR2 after shRNA knockdown (representative blot with densitometry normalized to GAPDH; n = 3; unpaired two−tailed t test). **(B, C)** CCK-8 assays showing proliferation capacity in U87 and U251 cells with IFNAR2 knockdown, n=3. **(D, E)** Clonogenic potential of IFNAR2-deficient U87 and U251 cells, n=3. **(F–I)** Transwell migration and invasion assays demonstrating impaired motility in IFNAR2-knockdown cells, cells/field; n=3. **(J)** Histological analyses of orthotopic glioma shNC, shIFNAR2#1, and shIFNAR2#2 groups. **(K)** Quantification of orthotopic xenograft volumes. **(L)** KM curves of glioma-bearing mice. **P < 0.01, ***P < 0.001; #P < 0.05, ##P < 0.01, ### P< 0.001.

We then conducted a series of experiments to examine the role of IFNAR2 in glioma pathology. Initial investigations demonstrated that IFNAR2 suppression markedly attenuated proliferative capacity, invasive potential, and migratory activity in both U87 and U251 glioma cell models (**Figures 11A–I**).

We then utilized a U87 orthotopic xenograft mouse model to further assess the effects of IFNAR2 gene inhibition on tumor development in mice. The results indicated that, in comparison with the control group, mice in the IFNAR2 knockdown group exhibited a significant reduction in tumor volume and prolonged survival time within 30 days (**Figures 11J–L**). Extensive experimental results have verified the fundamental impact of IFNAR2 on fostering tumor proliferation.

### Pan-cancer applicability of MRRG risk models

We systematically evaluated the prognostic utility of MRRG risk scores across 33 TCGA pan-cancer expression profiles. Cox regression analyses were estimated to calculate HRs with 95%CIs for individual types, followed by random-effects meta-analysis. The integrated results demonstrated a significant association between elevated MRRG scores and adverse pan-cancer outcomes (pooled OR = 1.19, 95%CI [1.07-1.31], **Supplementary Figure S7**), highlighting its potential as a cross-cancer prognostic biomarker.

## Discussion

Gliomas, the most prevalent primary malignant tumors in the central nervous system, are classified into LGG and GBM, with high-grade subtypes characterized by aggressive progression and dismal prognosis (1, 2). Regardless of advancements in surgical and pharmacological interventions, patient outcomes remain suboptimal due to the immunosuppressive tumor microenvironment, compromised blood-brain barrier permeability, acquired therapeutic resistance, and frequent recurrence (35, 36). Thus, developing novel therapeutic strategies to enhance targeted treatment responsiveness and establishing precision prognostic surveillance systems constitute critical unmet needs in contemporary neuro-oncology. Mitophagy, a crucial regulatory mechanism for mitochondrial quality control and homeostasis, has been implicated in tumor progression through metabolic reprogramming, ROS homeostasis regulation, and immune evasion (6–8, 37). However, the systemic relevance of mitophagy receptors—molecular initiators of this process—in gliomas remains elusive. In the job, we generated a robust mitophagy receptor-related prognostic model through multi-omics integration, which not only enhanced gliomas risk stratification accuracy but also demonstrated pan-cancer predictive potential. Furthermore, we uncovered IFNAR2 as a critical regulator driving glioma genesis.

This study revealed significant differential expression patterns of mitophagy receptor genes between tumor and normal tissues. The mitophagy receptor enrichment scores demonstrated strong associations with adverse prognosis. Through integrative WGCNA co-expression network analysis and single-cell AUCell functional scoring, we systematically filtered out prognostic gene clusters and established the novel MRRG prognostic risk model using Lasso analysis. Cox regression analysis confirmed the independent prognostic significance of MRRG risk scores. The prognostic capacity outperformed existing glioma prognostic models (38–40). Given the universal hallmarks of metabolic reprogramming and immune evasion in malignancies, pan-cancer meta-analysis further substantiated the cross-cancer prognostic applicability of the MRRG model, offering a unified framework to address prognostic heterogeneity across tumor types.

Leveraging the Cell-chat computational framework, we deciphered intercellular signaling networks across single-cell subpopulations, identifying pronounced activation of MHC-I, MIF, and SPP1 immune axes. Mechanistically: Malignant cells attenuate antigen presentation systems via the MHC-I signaling axis, compromising CD8+ T cell-mediated immunosurveillance (41–43); MIF signaling constrains microglial differentiation toward pro-inflammatory states (29); SPP1+ TAMs orchestrate T cell exhaustion (44, 45). The above implied that a sophisticated immunological evasion system operated in the microenvironment of gliomas. Integrative computational analysis revealed a prototypical immunosuppressive signature in high-risk cohorts, characterized by dominant M2-polarized macrophage infiltration, coupled with coordinated expansion of quiescent NK cells and resting CD4+T cells. Notably, this subgroup exhibited systemic depletion of Tregs, Tcm, and Tfh, collectively indicative of an immune-exhausted microenvironment. As immunotherapy represents an emerging approach in cancer treatment (3, 46), this investigation further investigated the relationship between MRRG risk scores and immunotherapy feasibility. Primarily, the significant positive correlation between TIDE scores and risk scores suggested that high-risk patients may undergo reduced immunotherapy response rates due to immune escape mechanisms (47). Then, the strong positive correlation between risk scores and EMT-scores implied a potential propensity for primary drug resistance in this cohort (26). The current standard of care for gliomas involves surgical resection followed by adjuvant chemotherapy, but therapeutic efficacy varies due to tumor heterogeneity, with a high probability of drug resistance (48). Drug resistance evaluation revealed an inverse relationship between the sensitivity of standard chemotherapeutic agents (e.g., TMZ) and risk scores. Collectively, these findings demonstrate that the MRRG risk scoring system provides multidimensional molecular insights for personalized glioma treatment, holding potential translational significance for clinical applications.

Emerging findings highlight that interferon significantly improves the clinical prognosis of glioma patients by integrating treatment strategies such as standard chemotherapy as well as nanocarrier-based drug delivery systems (49, 50). As the core subunit of type I IFN receptor, IFNAR2 orchestrates IFN signaling transduction and plays critical roles in tumorigenesis, necessitating comprehensive elucidation of its biological implications in glioma pathogenesis (51).

This study established IFNAR2 as a prognostic risk factor in gliomas through integrative transcriptomic analyses. Mendelian randomization and colocalization analyses further validated IFNAR2 as a hereditary susceptibility gene for brain tumorigenesis, identifying its pivotal regulatory SNP locus. Functional *in vitro* and *in vivo* experiments demonstrated IFNAR2’s role in driving gliomas’ malignant progression via cell migration and invasion.

This study began with the fundamental component of signal transduction—receptors—and focuses on mitophagy, a core mechanism for maintaining cellular homeostasis, to innovatively investigate the biological implications of mitophagy receptors on gliomas. We developed a robust mitophagy receptor-related risk model, which is rigorously validated as a powerful prognostic feature for gliomas. Elevated risk scores significantly related to adverse outcomes and an immunosuppressive microenvironment characterized by M2 macrophage polarization and T cell exhaustion. Additionally, the MRRG score accurately predicted therapeutic responsiveness. Finally, through genetic analyses and *in vitro*/*in vivo* functional experiments, we preliminarily delineated the multifaceted regulatory interventions of IFNAR2 in tumor initiation and progression.

Our work is limited by its retrospective reliance on heterogeneous public RNA−seq cohorts, which may harbor batch effects despite normalization. Sample diversity is restricted (few pediatric or rare molecular subtypes), potentially limiting generalizability. Single−cell analyses involve a small number of specimens without spatial context. Drug/immunotherapy responsiveness is inferred computationally, not clinically validated. Functional experiments focused only on IFNAR2, leaving other MRRGs unexplored mechanistically. Prospective, multi−center, multi−omic and broader functional validation will be necessary before clinical translation.

Collectively, this study innovatively proposes a mitophagy receptor-related risk signature as a prognostic stratification tool for gliomas, while identifying IFNAR2 as a novel therapeutic target.

## Conclusion

We developed and externally validated a prognostic model based on mitophagy MRRGs, which demonstrated superior prognostic stratification capabilities compared to conventional clinical markers in glioma. By integrating bulk transcriptomic and single-cell data along with preliminary functional validation of IFNAR2, our findings suggest that mitophagy receptor signaling may modulate immune functions within the tumor microenvironment, such as macrophage polarization and T cell activity. Clinically, this model holds potential value in three aspects: (i) supporting individualized prognostic evaluation; (ii) assisting in imaging surveillance planning; and (iii) providing a basis for patient stratification in future trials involving immunotherapy. The identification of IFNAR2 and other MRRG components as potential biomarkers lays a groundwork for further research into combined targeting strategies involving mitophagy and immune modulation. Although prospective, multi-center, and higher-resolution studies are necessary before clinical application, the current study indicates that the MRRG signature offers a novel perspective for prognostic stratification and informs the design of future combination therapies.

## Data Availability

The original contributions presented in the study are included in the article/**Supplementary Material**. Further inquiries can be directed to the corresponding authors.
