# Supplementary material for: Multi-omics insights into the role of mitophagy receptor-related genes in glioma prognosis and immune microenvironment remodeling
Source: Front Immunol. 2025 Oct 15;16:1672678. doi: 10.3389/fimmu.2025.1672678 (PMC12568465; doi:10.3389/fimmu.2025.1672678)
Supplement: Supplementary file 1 [file DataSheet1.pdf]

Supplementary materials

Figure S1: Identification and analysis of single-cell subpopulations.

Figure S2: Cell-Chat analyses.

Figure S3: Expression of MRRG signature model genes at the single-cell level.

Figure S4: Expression of MRRG risk genes at the single-cell level.

Figure S5: Reshaping of the immunological microenvironment by MRRG signatures.

Figure S6: Mendelian randomization analysis results of IFNAR2 and brain tumors.

Figure S7: Pan-cancer meta forest plot.

Table S1: Summary of 16 mitophagy receptor genes and their literature sources

Table S2: Univariate and multivariate Cox regression analyses based on MRRG risk scores and other clinicopathological variables.

Table S3: The gene set enrichment analysis (GSEA) results based on the MRRG risk scores.

Table S4: Correlation results between immune checkpoints and MRRG risk scores.

Table S5: Univariate and multivariate Cox regression analyses based on IFNAR2 expression levels and other clinicopathological variables.

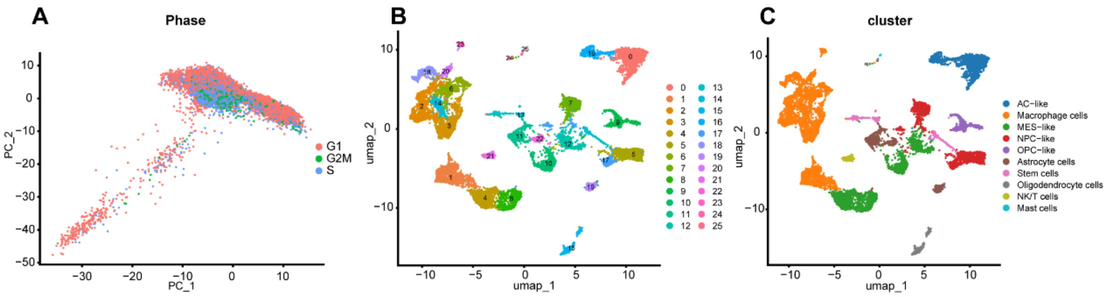

Figure S1: Identification and analysis of single-cell subpopulations. (A) PCA plot of the cell cycle. (B-C) UMAP plot showing the single-cell dataset divided into 26 subpopulations and identified as 10 cell clusters.

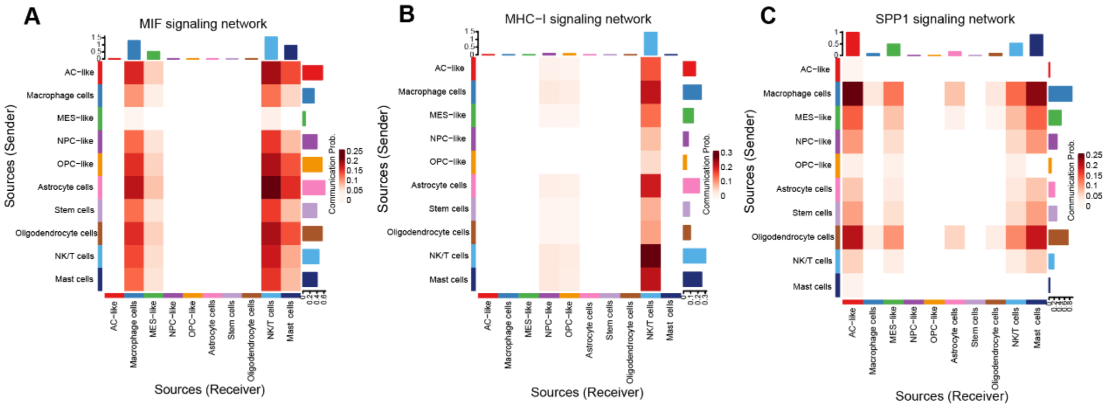

Figure S2: Cell-Chat analyses. (A-C) Visualization of communication between MIF, MHC-I and SPP1 pathway cell subpopulations via heatmaps.

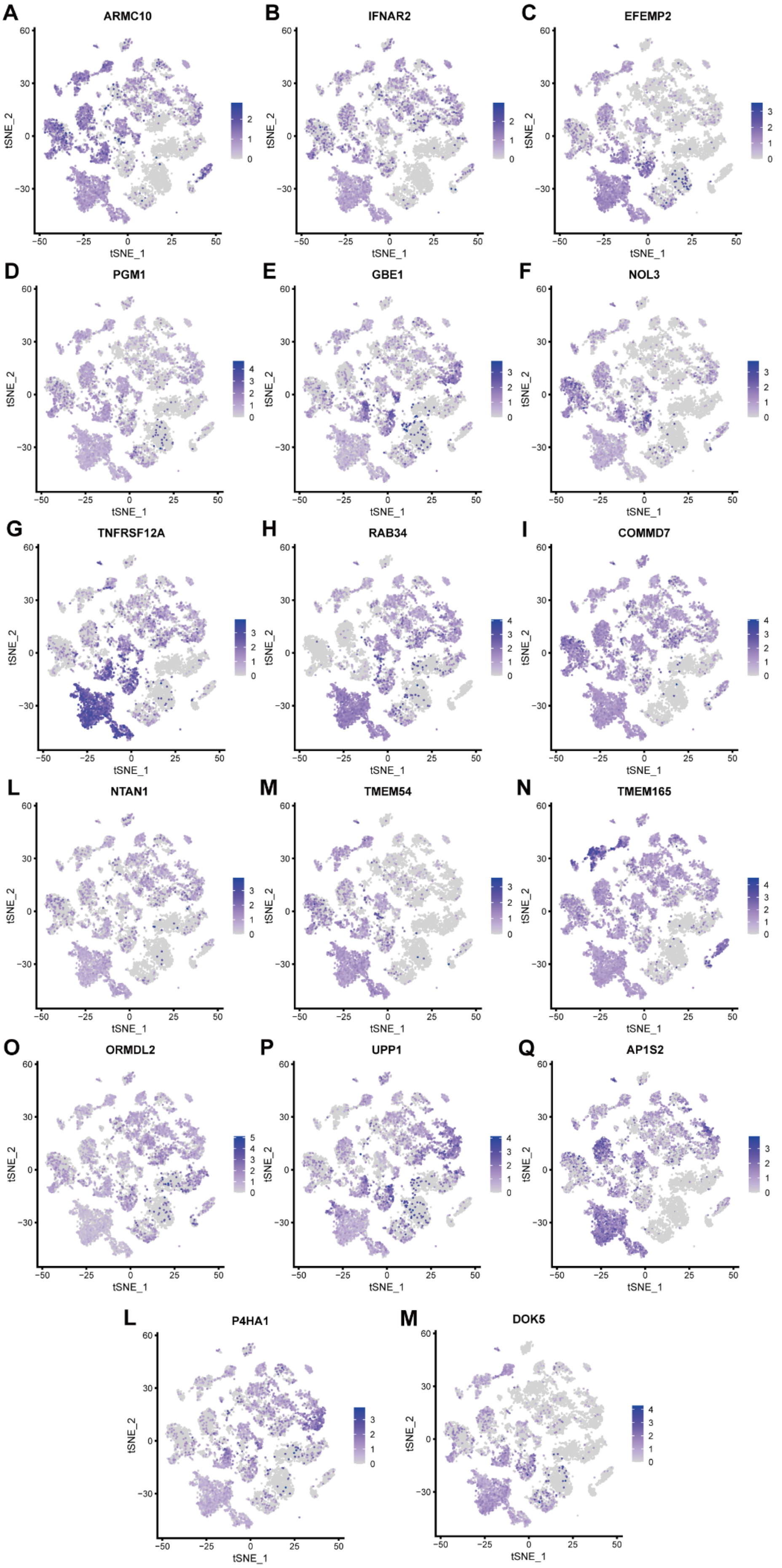
A
ARMC10
B
IFNAR2
C
EFEMP2
D
PGM1
E
GBE1
F
NOL3
G
TNFRSF12A
H
RAB34
I
COMMD7
L
NTAN1
M
TMEM54
N
TMEM165
O
ORMDL2
P
UPP1
Q
AP1S2
L
P4HA1
M
DOK5
tSNE_1
tSNE_2

Figure S3: Expression of MRRG signature model genes at the single-cell level.

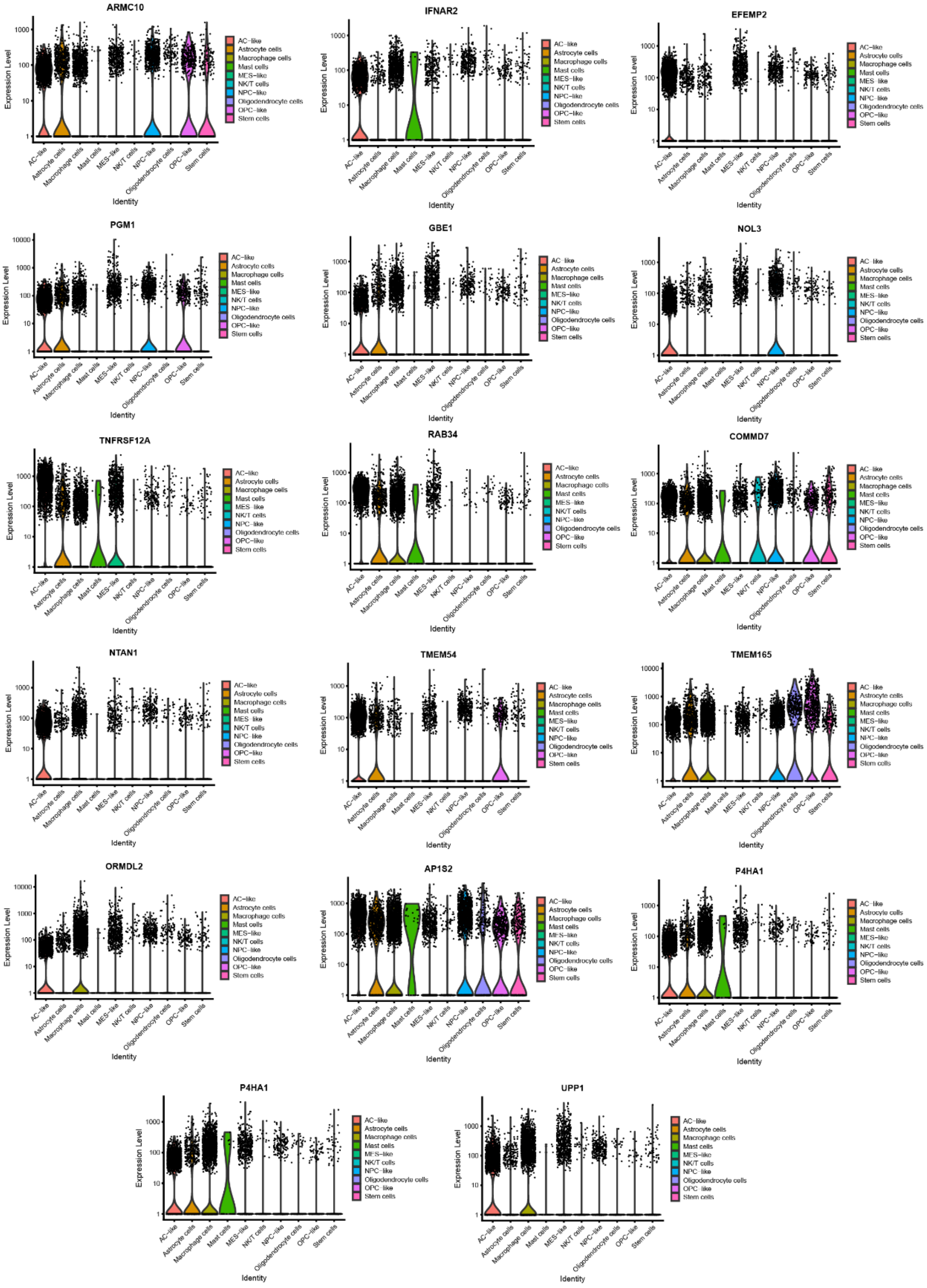

Figure S4: Expression of MRRG risk genes at the single-cell level.

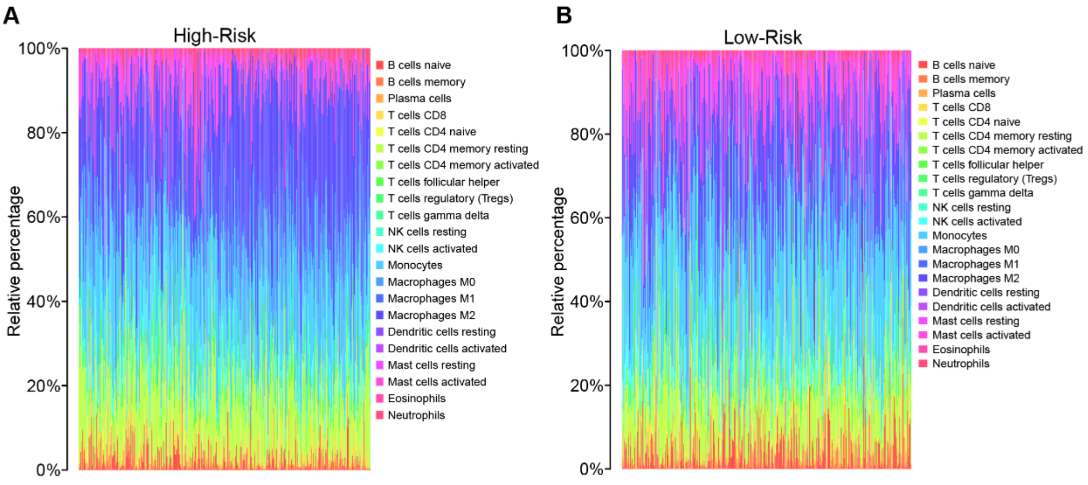

Figure S5: Reshaping of the immunological microenvironment by MRRG signatures. (A-B) Compound bar graphs of leukocyte subset proportions in two MRRG risk groups.

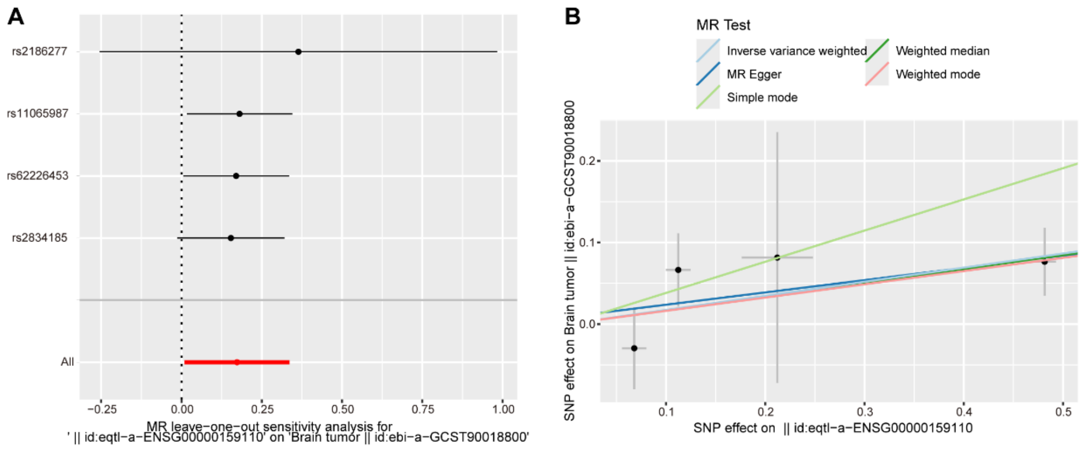

Figure S6: Mendelian randomization analysis results of IFNAR2 and brain tumors.

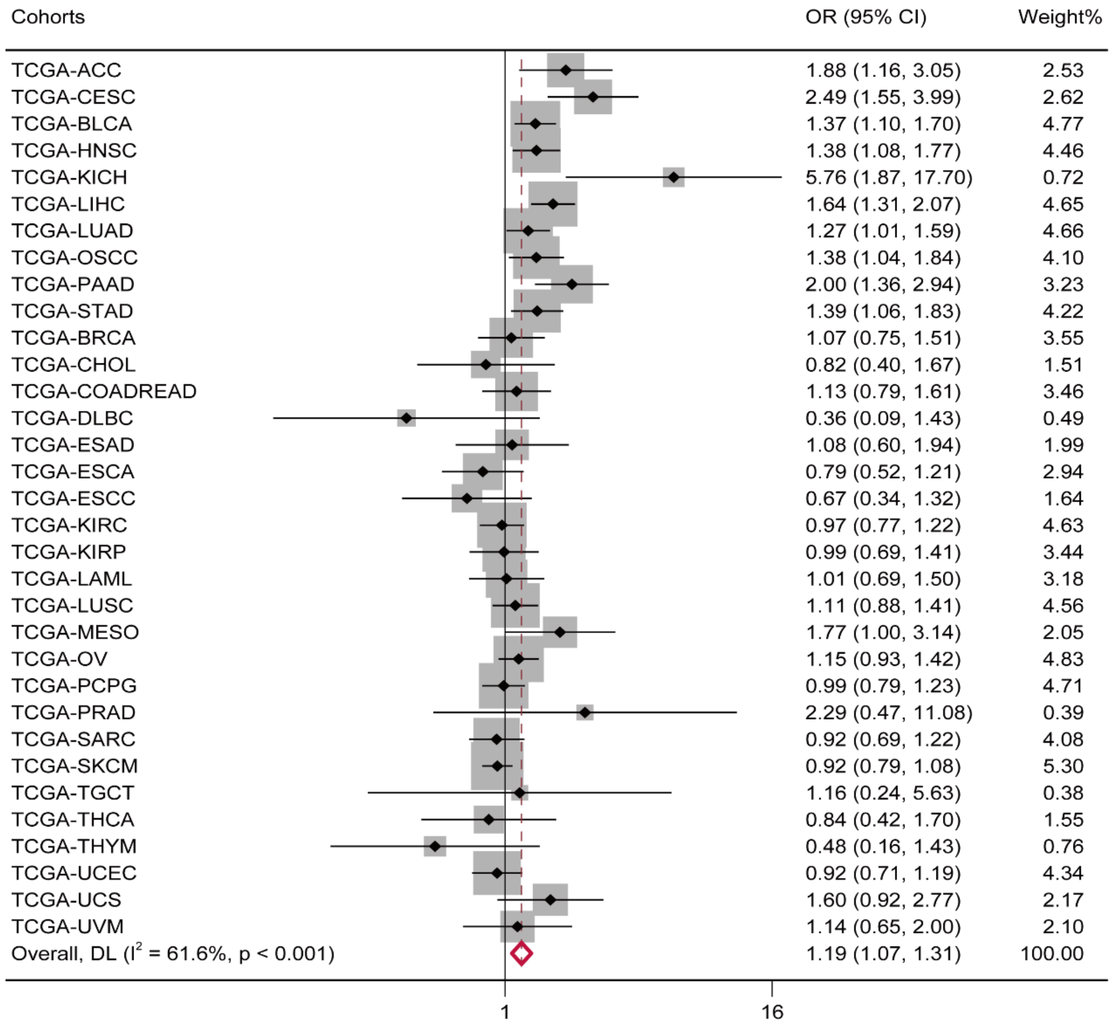

NOTE: Weights are from random-effects model

Figure S7: Pan-cancer meta forest plot.

Table S1: Summary of Sixteen Mitophagy Receptor Genes and Their Literature Sources

Gene Symbol	Gene Name	Key Supporting Reference(s)
FUNDC1	FUN14 Domain Containing 1	[9]Zhang Q, Chen C, Ma Y, Yan X, Lai N, Wang H, et al. PGAM5 interacts with and maintains BNIP3 to license cancer-associated muscle wasting. Autophagy. 2024;20(10):2205-20.<br><br>[10]Lim Y, Berry B, Viteri S, McCall M, Park EC, Rongo C, et al. FNDC-1-mediated mitophagy and ATFS-1 coordinate to protect against hypoxia-reoxygenation. Autophagy. 2021;17(11):3389-401.<br><br>[11]Cen X, Chen Y, Xu X, Wu R, He F, Zhao Q, et al. Pharmacological targeting of MCL-1 promotes mitophagy and improves disease pathologies in an Alzheimer's disease mouse model. Nature communications. 2020;11(1):5731.<br><br>[12] Wang ZT, Lu MH, Zhang Y, Ji WL, Lei L, Wang W, et al. Disrupted-in-schizophrenia-1 protects synaptic plasticity in a transgenic mouse model of Alzheimer's disease as a mitophagy receptor. Aging cell. 2019;18(1):e12860.<br><br>[13] Sulkshane P, Ram J, Thakur A, Reis N, Kleifeld O, Glickman MH. Ubiquitination and receptor-mediated mitophagy converge to eliminate oxidation-damaged mitochondria during hypoxia. Redox biology. 2021;45:102047.<br><br>[14] Marinković M, Šprung M, Novak I. Dimerization of mitophagy receptor BNIP3L/NIX is essential for recruitment of autophagic machinery. Autophagy. 2021;17(5):1232-43.<br><br>[15] Strappazzon F, Di Rita A, Peschiaroli A, Leoncini PP, Locatelli F, Melino G, et al. HUWE1 controls MCL1 stability to unleash AMBRA1-induced mitophagy. Cell death and differentiation. 2020;27(4):1155-68.<br><br>[18] Xu X, Zhang Y, Cheng H, Zhou R. SPATA33 functions as a mitophagy receptor in mammalian germline. Autophagy. 2021;17(5):1284-6.
MCL1	MCL1 Apoptosis Regulator,	[11] Cen X, Chen Y, Xu X, Wu R, He F, Zhao Q, et al. Pharmacological targeting of MCL-1 promotes mitophagy and improves disease pathologies in an Alzheimer's disease mouse model. Nature communications.

	BCL2 Family Member	2020;11(1):5731.<br><br>[21] Cen X, Xu X, Xia H. Targeting MCL1 to induce mitophagy is a potential therapeutic strategy for Alzheimer disease. Autophagy. 2021;17(3):818-9.
SQSTM1	Sequestosome 1	[11]Cen X, Chen Y, Xu X, Wu R, He F, Zhao Q, et al. Pharmacological targeting of MCL-1 promotes mitophagy and improves disease pathologies in an Alzheimer's disease mouse model. Nature communications. 2020;11(1):5731.<br><br>[12] Wang ZT, Lu MH, Zhang Y, Ji WL, Lei L, Wang W, et al. Disrupted-in-schizophrenia-1 protects synaptic plasticity in a transgenic mouse model of Alzheimer's disease as a mitophagy receptor. Aging cell. 2019;18(1):e12860.<br><br>[13] Sulkshane P, Ram J, Thakur A, Reis N, Kleifeld O, Glickman MH. Ubiquitination and receptor-mediated mitophagy converge to eliminate oxidation-damaged mitochondria during hypoxia. Redox biology. 2021;45:102047.
NBR1	NBR1 Autophagy Cargo Receptor	[11] Cen X, Chen Y, Xu X, Wu R, He F, Zhao Q, et al. Pharmacological targeting of MCL-1 promotes mitophagy and improves disease pathologies in an Alzheimer's disease mouse model. Nature communications. 2020;11(1):5731.<br><br>[12] Wang ZT, Lu MH, Zhang Y, Ji WL, Lei L, Wang W, et al. Disrupted-in-schizophrenia-1 protects synaptic plasticity in a transgenic mouse model of Alzheimer's disease as a mitophagy receptor. Aging cell. 2019;18(1):e12860.<br><br>[13] Sulkshane P, Ram J, Thakur A, Reis N, Kleifeld O, Glickman MH. Ubiquitination and receptor-mediated mitophagy converge to eliminate oxidation-damaged mitochondria during hypoxia. Redox biology. 2021;45:102047.<br><br>[17] Abudu YP, Pankiv S, Mathai BJ, Lamark T, Johansen T, Simonsen A. NIPSNAP1 and NIPSNAP2 act as "eat me" signals to allow sustained recruitment of autophagy receptors during mitophagy. Autophagy. 2019;15(10):1845-7.
AMBRA1	Autophagy And Beclin 1	[11] Cen X, Chen Y, Xu X, Wu R, He F, Zhao Q, et al. Pharmacological targeting of MCL-1 promotes mitophagy and improves disease pathologies

	Regulator 1	in an Alzheimer's disease mouse model. Nature communications. 2020;11(1):5731.<br><br>[12] Wang ZT, Lu MH, Zhang Y, Ji WL, Lei L, Wang W, et al. Disrupted-in-schizophrenia-1 protects synaptic plasticity in a transgenic mouse model of Alzheimer's disease as a mitophagy receptor. Aging cell. 2019;18(1):e12860.<br><br>[14] Marinković M, Šprung M, Novak I. Dimerization of mitophagy receptor BNIP3L/NIX is essential for recruitment of autophagic machinery. Autophagy. 2021;17(5):1232-43.<br><br>[15] Strappazzon F, Di Rita A, Peschiaroli A, Leoncini PP, Locatelli F, Melino G, et al. HUWE1 controls MCL1 stability to unleash AMBRA1-induced mitophagy. Cell death and differentiation. 2020;27(4):1155-68.<br><br>[20] Dagar N, Kale A, Steiger S, Anders HJ, Gaikwad AB. Receptor-mediated mitophagy: An emerging therapeutic target in acute kidney injury. Mitochondrion. 2022;66:82-91.
BCL2L13	BCL2 Like 13	[11] Cen X, Chen Y, Xu X, Wu R, He F, Zhao Q, et al. Pharmacological targeting of MCL-1 promotes mitophagy and improves disease pathologies in an Alzheimer's disease mouse model. Nature communications. 2020;11(1):5731.<br><br>[14] Marinković M, Šprung M, Novak I. Dimerization of mitophagy receptor BNIP3L/NIX is essential for recruitment of autophagic machinery. Autophagy. 2021;17(5):1232-43.<br><br>[16] Otsu K, Murakawa T, Yamaguchi O. BCL2L13 is a mammalian homolog of the yeast mitophagy receptor Atg32. Autophagy. 2015;11(10):1932-3.<br><br>[18]Xu X, Zhang Y, Cheng H, Zhou R. SPATA33 functions as a mitophagy receptor in mammalian germline. Autophagy. 2021;17(5):1284-6.
SMURF1	SMAD Specific E3 Ubiquitin Protein Ligase 1	[20] Dagar N, Kale A, Steiger S, Anders HJ, Gaikwad AB. Receptor-mediated mitophagy: An emerging therapeutic target in acute kidney injury. Mitochondrion. 2022;66:82-91.
NIPSNAP1	Nipsnap Homolog 1	[17] Abudu YP, Pankiv S, Mathai BJ, Lamark T, Johansen T, Simonsen A. NIPSNAP1 and NIPSNAP2 act as "eat me" signals to allow sustained recruitment of autophagy receptors during mitophagy. Autophagy.

		2019;15(10):1845-7.
NIPSNAP2	Nipsnap Homolog 2	[17] Abudu YP, Pankiv S, Mathai BJ, Lamark T, Johansen T, Simonsen A. NIPSNAP1 and NIPSNAP2 act as "eat me" signals to allow sustained recruitment of autophagy receptors during mitophagy. Autophagy. 2019;15(10):1845-7.
SPATA33	Spermatogenesis Associated 33	[18] Xu X, Zhang Y, Cheng H, Zhou R. SPATA33 functions as a mitophagy receptor in mammalian germline. Autophagy. 2021;17(5):1284-6.
MTFP1	Mitochondrial Fission Process 1	[19] Panigrahi DP, Praharaj PP, Behera BP, Patra S, Patil S, Patro BS, et al. The inner mitochondrial membrane fission protein MTP18 serves as a mitophagy receptor to prevent apoptosis in oral cancer. Journal of cell science. 2023;136(13).
BNIP3	BCL2 Interacting Protein 3	[13] Sulkshane P, Ram J, Thakur A, Reis N, Kleifeld O, Glickman MH. Ubiquitination and receptor-mediated mitophagy converge to eliminate oxidation-damaged mitochondria during hypoxia. Redox biology. 2021;45:102047.<br>[14] Marinković M, Šprung M, Novak I. Dimerization of mitophagy receptor BNIP3L/NIX is essential for recruitment of autophagic machinery. Autophagy. 2021;17(5):1232-43.<br>[15] Strappazzon F, Di Rita A, Peschiaroli A, Leoncini PP, Locatelli F, Melino G, et al. HUWE1 controls MCL1 stability to unleash AMBRA1-induced mitophagy. Cell death and differentiation. 2020;27(4):1155-68.<br>[18] Xu X, Zhang Y, Cheng H, Zhou R. SPATA33 functions as a mitophagy receptor in mammalian germline. Autophagy. 2021;17(5):1284-6.<br>[20] Dagar N, Kale A, Steiger S, Anders HJ, Gaikwad AB. Receptor-mediated mitophagy: An emerging therapeutic target in acute kidney injury. Mitochondrion. 2022;66:82-91.
BNIP3L	BCL2 Interacting Protein 3 Like	[11] Cen X, Chen Y, Xu X, Wu R, He F, Zhao Q, et al. Pharmacological targeting of MCL-1 promotes mitophagy and improves disease pathologies in an Alzheimer's disease mouse model. Nature communications. 2020;11(1):5731.<br>[12] Wang ZT, Lu MH, Zhang Y, Ji WL, Lei L, Wang W, et al. Disrupted-in-schizophrenia-1 protects synaptic plasticity in a transgenic mouse model of Alzheimer's disease as a mitophagy receptor. Aging cell.

		2019;18(1):e12860.<br><br>[13] Sulkshane P, Ram J, Thakur A, Reis N, Kleifeld O, Glickman MH. Ubiquitination and receptor-mediated mitophagy converge to eliminate oxidation-damaged mitochondria during hypoxia. Redox biology. 2021;45:102047.<br><br>[14] Marinković M, Šprung M, Novak I. Dimerization of mitophagy receptor BNIP3L/NIX is essential for recruitment of autophagic machinery. Autophagy. 2021;17(5):1232-43.<br><br>[15] Strappazzon F, Di Rita A, Peschiaroli A, Leoncini PP, Locatelli F, Melino G, et al. HUWE1 controls MCL1 stability to unleash AMBRA1-induced mitophagy. Cell death and differentiation. 2020;27(4):1155-68.<br><br>[18] Xu X, Zhang Y, Cheng H, Zhou R. SPATA33 functions as a mitophagy receptor in mammalian germline. Autophagy. 2021;17(5):1284-6.<br><br>[20] Dagar N, Kale A, Steiger S, Anders HJ, Gaikwad AB. Receptor-mediated mitophagy: An emerging therapeutic target in acute kidney injury. Mitochondrion. 2022;66:82-91.
FKBP8	FKBP Prolyl Isomerase 8	[13] Sulkshane P, Ram J, Thakur A, Reis N, Kleifeld O, Glickman MH. Ubiquitination and receptor-mediated mitophagy converge to eliminate oxidation-damaged mitochondria during hypoxia. Redox biology. 2021;45:102047.<br><br>[14] Marinković M, Šprung M, Novak I. Dimerization of mitophagy receptor BNIP3L/NIX is essential for recruitment of autophagic machinery. Autophagy. 2021;17(5):1232-43.<br><br>[18] Xu X, Zhang Y, Cheng H, Zhou R. SPATA33 functions as a mitophagy receptor in mammalian germline. Autophagy. 2021;17(5):1284-6.<br><br>[20] Dagar N, Kale A, Steiger S, Anders HJ, Gaikwad AB. Receptor-mediated mitophagy: An emerging therapeutic target in acute kidney injury. Mitochondrion. 2022;66:82-91.
PHB2	Prohibitin 2	[9] Zhang Q, Chen C, Ma Y, Yan X, Lai N, Wang H, et al. PGAM5 interacts with and maintains BNIP3 to license cancer-associated muscle wasting. Autophagy. 2024;20(10):2205-20.<br><br>[11] Cen X, Chen Y, Xu X, Wu R, He F, Zhao Q, et al. Pharmacological

		targeting of MCL-1 promotes mitophagy and improves disease pathologies in an Alzheimer's disease mouse model. Nature communications. 2020;11(1):5731.<br><br>[14] Marinković M, Šprung M, Novak I. Dimerization of mitophagy receptor BNIP3L/NIX is essential for recruitment of autophagic machinery. Autophagy. 2021;17(5):1232-43.<br><br>[15] Strappazzon F, Di Rita A, Peschiaroli A, Leoncini PP, Locatelli F, Melino G, et al. HUWE1 controls MCL1 stability to unleash AMBRA1-induced mitophagy. Cell death and differentiation. 2020;27(4):1155-68.<br><br>[20] Dagar N, Kale A, Steiger S, Anders HJ, Gaikwad AB. Receptor-mediated mitophagy: An emerging therapeutic target in acute kidney injury. Mitochondrion. 2022;66:82-91.<br><br>[22] Li J, Shi X, Xu J, Wang K, Hou F, Luan X, et al. Aldehyde Dehydrogenase 2 Lactylation Aggravates Mitochondrial Dysfunction by Disrupting PHB2 Mediated Mitophagy in Acute Kidney Injury. Advanced science (Weinheim, Baden-Wurttemberg, Germany). 2025;12(8):e2411943.
TAX1BP1	Tax1 Binding Protein 1	[12] Wang ZT, Lu MH, Zhang Y, Ji WL, Lei L, Wang W, et al. Disrupted-in-schizophrenia-1 protects synaptic plasticity in a transgenic mouse model of Alzheimer's disease as a mitophagy receptor. Aging cell. 2019;18(1):e12860.<br><br>[13] Sulkshane P, Ram J, Thakur A, Reis N, Kleifeld O, Glickman MH. Ubiquitination and receptor-mediated mitophagy converge to eliminate oxidation-damaged mitochondria during hypoxia. Redox biology. 2021;45:102047.<br><br>[17] Abudu YP, Pankiv S, Mathai BJ, Lamark T, Johansen T, Simonsen A. NIPSNAP1 and NIPSNAP2 act as "eat me" signals to allow sustained recruitment of autophagy receptors during mitophagy. Autophagy. 2019;15(10):1845-7.

Table S2: Univariate and multivariate Cox regression analyses based on MRRG risk scores and other clinicopathological variables.

Characteristics	Total(N)	Univariate analysis		Multivariate analysis	
		Hazard ratio (95% CI)	P value	Hazard ratio (95% CI)	P value
Risk-Scores	678				
Low	344	Reference		Reference	
High	334	7.838 (5.719 - 10.743)	**< 0.001**	2.663 (1.700 - 4.173)	**< 0.001**
Age	678	1.050 (1.041 - 1.060)	**< 0.001**	1.030 (1.020 - 1.040)	**< 0.001**
WHO Grade	678				
G2&G3	409	Reference		Reference	
G4	269	8.557 (6.473 - 11.312)	**< 0.001**	1.951 (1.063 - 3.582)	**0.031**
IDH Mutation	678				
Mutant	436	Reference		Reference	
WT	242	8.909 (6.812 - 11.653)	**< 0.001**	1.857 (1.079 - 3.197)	**0.026**
1p/19q codel	678				
non-codel	509	Reference		Reference	
codel	169	0.226 (0.147 - 0.348)	**< 0.001**	0.738 (0.439 - 1.240)	0.252

Table S3: The gene set enrichment analysis (GSEA) results based on the MRRG risk scores.

Gene set name	NES	P.val
GO_NEURON_DEVELOPMENT	2.851	3.01E-08
GO_CALCIUM_ION_REGULATED_EXOCYTOSIS	2.720	3.67E-05
GO_VESICLE_MEDIATED_TRANSPORT_IN_SYNAPSE	2.681	3.66E-05
GO_TRANSMEMBRANE_TRANSPORTER_ACTIVITY	2.622	1.39E-06
GO_LEUKOCYTE_MEDIATED_IMMUNITY	-2.744	5.23E-09
GO_REGULATION_OF_IMMUNE_RESPONSE	-2.763	3.90E-08
GO_IMMUNE_EFFECTOR_PROCESS	-2.938	1.00E-10
GO_CYTOKINE_MEDIATED_SIGNALING_PATHWAY	-2.992	4.14E-10
KEGG_CALCIUM_SIGNALING_PATHWAY	2.749	1.52E-05
KEGG_NEUROACTIVE_LIGAND_RECEPTOR_INTERACTION	2.683	2.13E-05
KEGG_LONG_TERM_POTENTIATION	2.098	< 0.005
KEGG_JAK_STAT_SIGNALING_PATHWAY	-2.063	< 0.005
KEGG_CELL_CYCLE	-2.199	< 0.001
KEGG_CYTOKINE_CYTOKINE_RECEPTOR_INTERACTION	-2.201	< 0.001
KEGG_FOCAL_ADHESION	-2.508	4.56E-06
KEGG_ECM_RECEPTOR_INTERACTION	-2.869	3.23E-07

Table S4: Correlation results between immune checkpoints and MRRG risk scores.

Gene	Correlation	P
CD28	0.529257646	< 0.001
CTLA4	0.400884417	< 0.001
PDCD1	0.595387627	< 0.001
TNFSF14	0.714453119	< 0.001
HLA.F	0.532065495	< 0.001
HLA.G	0.28555466	< 0.001
CD80	0.655340896	< 0.001
HLA.DMA	0.676160935	< 0.001
CD40	0.676071377	< 0.001
CD96	0.640610709	< 0.001

Table S5: Univariate and multivariate Cox regression analyses based on IFNAR2 expression levels and other clinicopathological variables.

Characteristics	Total(N)	Univariate analysis		Multivariate analysis	
		Hazard ratio (95% CI)	P value	Hazard ratio (95% CI)	P value
WHO grade	636				
G2&G3	468	Reference		Reference	
G4	168	9.538 (7.243 - 12.560)	< **0.001**	1.821 (1.221 - 2.714)	**0.003**
IDH Mutation	688				
WT	246	Reference		Reference	
Mutation	442	0.116 (0.089 - 0.151)	< **0.001**	0.341 (0.225 - 0.516)	< **0.001**
1p/19q codel	691				
Non-codel	520	Reference		Reference	
Codel	171	0.225 (0.147 - 0.346)	< **0.001**	0.524 (0.315 - 0.873)	**0.013**
Age	698	1.066 (1.057 - 1.076)	< **0.001**	1.035 (1.023 - 1.047)	< **0.001**
IFNAR2	698	8.424 (6.096 - 11.640)	< **0.001**	1.819 (1.119 - 2.957)	**0.016**
